# Predictive Synthesis of Copper Selenides Using a Multidimensional Phase Map Constructed with a Data-Driven Classifier

**DOI:** 10.1021/jacs.3c05490

**Published:** 2023-08-04

**Authors:** Emily M. Williamson, Zhaohong Sun, Bryce A. Tappan, Richard L. Brutchey

**Affiliations:** Department of Chemistry, University of Southern California, Los Angeles, California 90089, United States

## Abstract

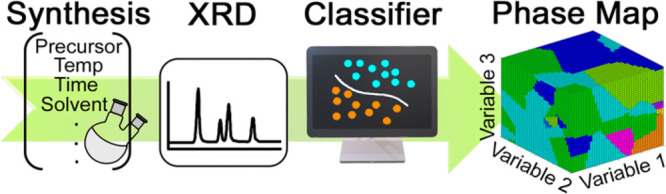

Copper selenides are an important family of materials with applications in catalysis, plasmonics, photovoltaics, and thermoelectrics. Despite being a binary material system, the Cu–Se phase diagram is complex and contains multiple crystal structures in addition to several metastable structures that are not found on the thermodynamic phase diagram. Consequently, the ability to synthetically navigate this complex phase space poses a significant challenge. We demonstrate that data-driven learning can successfully map this phase space in a minimal number of experiments. We combine soft chemistry (*chimie douce*) synthetic methods with multivariate analyses via classification techniques to enable predictive phase determination. A surrogate model was constructed with experimental data derived from a design matrix of four experimental variables: C–Se bond strength of the selenium precursor, time, temperature, and solvent composition. The reactions in the surrogate model resulted in 11 distinct phase combinations of copper selenide. These data were used to train a classification model that predicts the phase with 95.7% accuracy. The resulting decision tree enabled conclusions to be drawn about how the experimental variables affect the phase and provided prescriptive synthetic conditions for specific phase isolation. This guided the accelerated phase targeting in a minimum number of experiments of klockmannite CuSe, which could not be isolated in any of the reactions used to construct the surrogate model. The reaction conditions that the model predicted to synthesize klockmannite CuSe were experimentally validated, highlighting the utility of this approach.

## Introduction

The Materials Genome Initiative accelerated materials discovery through efforts such as the Materials Project, which combines supercomputing and density functional theory (DFT) to theoretically predict new materials and their properties before they are made.^1,2^ While this “materials by design” approach successfully identified vast numbers of materials with a wide range of targeted properties, a significant bottleneck now exists at the next step of the process: the Edisonian nature of materials synthesis. Unlike the vast majority of related reports from previous studies that develop approaches to discover new materials with specific properties,^3−10^ there is no robust predictive framework that can help map the reaction coordinate from precursors to the final crystalline solid when attempting to synthesize materials. Moreover, the compositions and structure types of crystalline inorganic solids are so disparate that it is exceptionally challenging to apply the lessons learned from one materials system to another. In fact, even “simple” binary systems can possess very complex thermodynamic phase diagrams that are synthetically difficult to navigate. For example, we invested multiple years of research to achieve phase control for binary nickel sulfide nanocrystals, where the key synthetic handle ended up being a nonintuitive, second-order interaction between the sulfur precursor and the surface ligand that distinguished between the Ni_3_S_4_, α-NiS, β-NiS, Ni_9_S_8_, and Ni_3_S_2_ phases.^11^ Additionally, Brock and co-workers studied the effects of “synthetic levers” on the synthesis of binary nickel phosphides, where intricate webs of numerous reaction pathways had to be disentangled in order to obtain the desired phase-pure products of Ni_12_P_5_, Ni_2_P, Ni_5_P_4_, and NiP_2_.^12,13^

Binary copper selenides are an important family of materials with applications in catalysis, plasmonics, photovoltaics, and thermoelectrics.^14−20^ These wide-ranging functional properties are a direct result of the rich and complex Cu–Se phase diagram that encompasses compositions spanning from Cu_2_Se to CuSe_2_ with multiple distinct crystal structures.^21^ The most common crystal structures reported in the literature are berzelianite Cu_2–*x*_Se (cubic, space group *Fm*3̅*m*), umangite Cu_3_Se_2_ (tetragonal, space group *P*4̅2_1_*m*), klockmannite CuSe (γ-CuSe, hexagonal, space group *P*6_3_/*mmc*, which transforms at lower temperatures into β-CuSe, orthorhombic, space group *Cmcm*), pyritic krutaite CuSe_2_ (cubic, space group *Pa*3̅), and marcasitic krutaite CuSe_2_ (orthorhombic, space group *Pnnm*).^22−27^ In recent years, several additional metastable copper selenide polymorphs that do not exist on thermodynamic phase diagrams have also been reported, including weissite-like Cu_2–*x*_Se (trigonal, space group *P*3̅*m*1) and wurtzite-like Cu_2–*x*_Se (hexagonal, space group *P*6_3_*mc*).^28−31^ In addition to their structural diversity eliciting an array of unique physicochemical properties, copper selenides have been found to function as essential binary intermediates in the synthesis of higher-order, multinary structures that adopt topologically related anion sublattices.^17^ For example, umangite Cu_3_Se_2_, which has a nearly hexagonal Se^2–^ sublattice, is a necessary intermediate in the synthesis of the metastable, wurtzite-like phases of CuInSe_2_, Cu_2_ZnSnSe_4_, and Cu_2_FeSnSe_4_.^32−34^

Traditional high-temperature solid-state techniques generally do not provide sufficient synthetic control to trap metastable products at ambient temperature and pressure, which makes navigating the complex phase diagrams for systems like Cu–Se difficult.^35,36^ This is because the solid-state synthesis of copper selenides relies on supplying the reaction vessel with an excess of energy to overcome the kinetic barriers of solid-state diffusion, which typically drives the formation of the most thermodynamically stable product, with only a few notable exceptions.^37−39^ Alternatively, *chimie douce* (or soft chemistry) methods increase the probability of isolating phases across the entire phase diagram because of the possibility of exerting kinetic control.^40^ The power of *chimie douce* lies in the expansiveness of its experimental variable space; however, this presents a significant challenge in and of itself. That is, the targeted synthesis of a single material can be difficult because of the large number of variables that must be controlled to obtain the phase purity. Consequently, *chimie douce* materials syntheses are traditionally developed via fragmented empirical knowledge of the underlying consequences of experimental variables.^41,42^ For this reason, achieving phase control in such syntheses can be tedious and laborious.

Executing the synthesis of a phase-pure material is traditionally done using the one-variable-at-a-time (OVAT) method, where only one variable is changed at a time, while all the other variables are held constant. In an experimental domain where *n* experimental variables create an *n*-dimensional design space, this one-dimensional approach is not only time and labor intensive, but inefficient in revealing potentially critical higher-order interactions between experimental variables and their effects on synthetic outcomes, such as phase. The inability to map a more complete picture of how the experimental variable space correlates to phase determination is limiting.^43^ One solution is to utilize data-driven learning to render synthetic phase maps that allow for rational targeting of materials within the high-dimensional variable space. Because phase is a categorical (or discrete) outcome, regression-based multivariate techniques like design of experiments (DoE) and response surface methodology (RSM) cannot be used because they require continuous outcomes.^44,45^ Deep learning techniques like convolutional neural networks can map the multidimensional variable space, but they require large data sets that are not feasible when novel chemistry is being employed and/or done in a low-throughput manner.^46,47^ On the other hand, a trained classification algorithm can handle both smaller data sets and categorical variables, making it a tractable solution to this problem.^48^

Herein, we combine *chimie douce* synthesis with multivariate analyses via data-driven classification to accelerate the predictive phase determination of copper selenides. After training and testing a classification algorithm on sparse reaction data, a synthetic phase map was constructed that encompasses an experimental variable space beyond the usual thermodynamic variables of composition and temperature. This is in contrast to the current examples seen in the literature,^3,4^ which use machine learning to predict thermodynamic phase diagrams, as the *phase maps* we construct capture both thermodynamic and kinetic information that guides the synthesis of materials beyond such thermodynamic levers. The resulting multidimensional phase map allows for an outcome to be predicted for a given set of experimental variables. We also show that the phase map can guide the synthesis of klockmannite CuSe, which was not isolable under any set of synthetic conditions used in the training data set. This is the first example of constructing a multidimensional phase map using data-driven classifiers, which streamlines the synthesis of distinct phases of copper selenide, including *terra incognita* metastable phases not found on the thermodynamic Cu–Se phase diagram.

## Results and Discussion

Our solution-phase synthesis of copper selenides is based on the relatively low-temperature reaction of Cu(oleate)_2_ with diorganyl diselenide precursors in oleylamine and 1-octadecene (ODE). Diorganyl diselenides (R–Se−Se−R, where R = alkyl, allyl, benzyl, or phenyl) have emerged over the past 15 years as versatile precursors for the kinetically controlled synthesis of metal selenides.^49−53^ The utility of these precursors stems from their programmable reactivity as a function of C–Se bond strength, which depends upon the identity of the functional group, as first proposed by Vela and co-workers.^54^ However, it has been shown by Macdonald and co-workers that this reactivity, along with the mechanisms of precursor conversion, concurrently depends upon the presence of Cu(oleate)_2_ and oleylamine,^42^ which is a C18 primary amine that acts as both a high-temperature solvent and reducing agent.^55,56^ This illustrates the high-dimensional nature of the chemistry, which complicates rational phase determination. We use a classification algorithm that identifies patterns in the reaction data to subsequently predict the copper selenide phase, or a combination of phases, for a given set of experimental variables. To accomplish this, a surrogate model was created that consists of reaction data sampled throughout the experimental space.^57,58^

### Construction of the Surrogate Model

A surrogate model provides the classification algorithm with the training and testing data required to map the patterns of the experimental variables and identify which variables are most important for phase determination. DoE screening and optimization matrices were utilized to construct the surrogate model. Despite the inability of the data to be modeled via regression because of its discrete nature, DoE provides an orthogonally balanced experimental sampling of the design space.^45,59^ This prevents the over- and under-sampling of any one region in the *n*-dimensional domain, which can occur when other techniques like random sampling are used.^44^

First, the variables to be investigated were chosen, and the synthetic bounds of the experimental space were set. The variables initially chosen for this investigation were: (1) C–Se precursor bond strength, (2) volumetric ratio of oleylamine to ODE, (3) temperature, and (4) time. To experimentally vary the C–Se bond strength, we chose two different diselenide precursors with C–Se bonds that differ by 22 kcal mol^–1^.^32^ The less reactive Ph_2_Se_2_ precursor has a stronger C–Se bond (BDE = 65 kcal mol^–1^), while the more reactive Bn_2_Se_2_ precursor has a weaker C–Se bond (BDE = 43 kcal mol^–1^). By limiting the diselenide precursors to those with two disparate C–Se bond strengths, we minimized the number of categorical variables in our screening, since categorical variables cause an exponential increase in the number of experiments required to satisfy a full screening design.^60^

The volumetric ratio of oleylamine to ODE was chosen because of the known influence of oleylamine on the decomposition mechanism of the diselenide precursors, in addition to its ability to act as a reducing agent for copper.^42,56^ This allows the amount of oleylamine to be varied continuously, while keeping the overall reaction concentration constant. Time and temperature were chosen due to their direct influence on the kinetics and thermodynamics of the reaction. The surrogate model was limited to these variables since the cost of increasing the number of variables and the corresponding number of required experiments outweighed the insight that would be gained. The bounds of the experimental space are provided in Table 1. The ranges of the continuous variables were chosen because values above or below these bounds rendered the reaction unsuccessful (no product), too time-consuming, or unfeasible due to synthetic limitations (e.g., temperatures significantly past solvent boiling points).

**Table 1:** Bounds of the Experimental Space

Bound	C–Se Bond Strength (kcal mol^–1^)	Oleylamine:ODE (*v*/*v*)	Temp (°C)	Time (min)
High	65	1:0	320	120
Low	43	1:20	170	1

**Figure 1:**
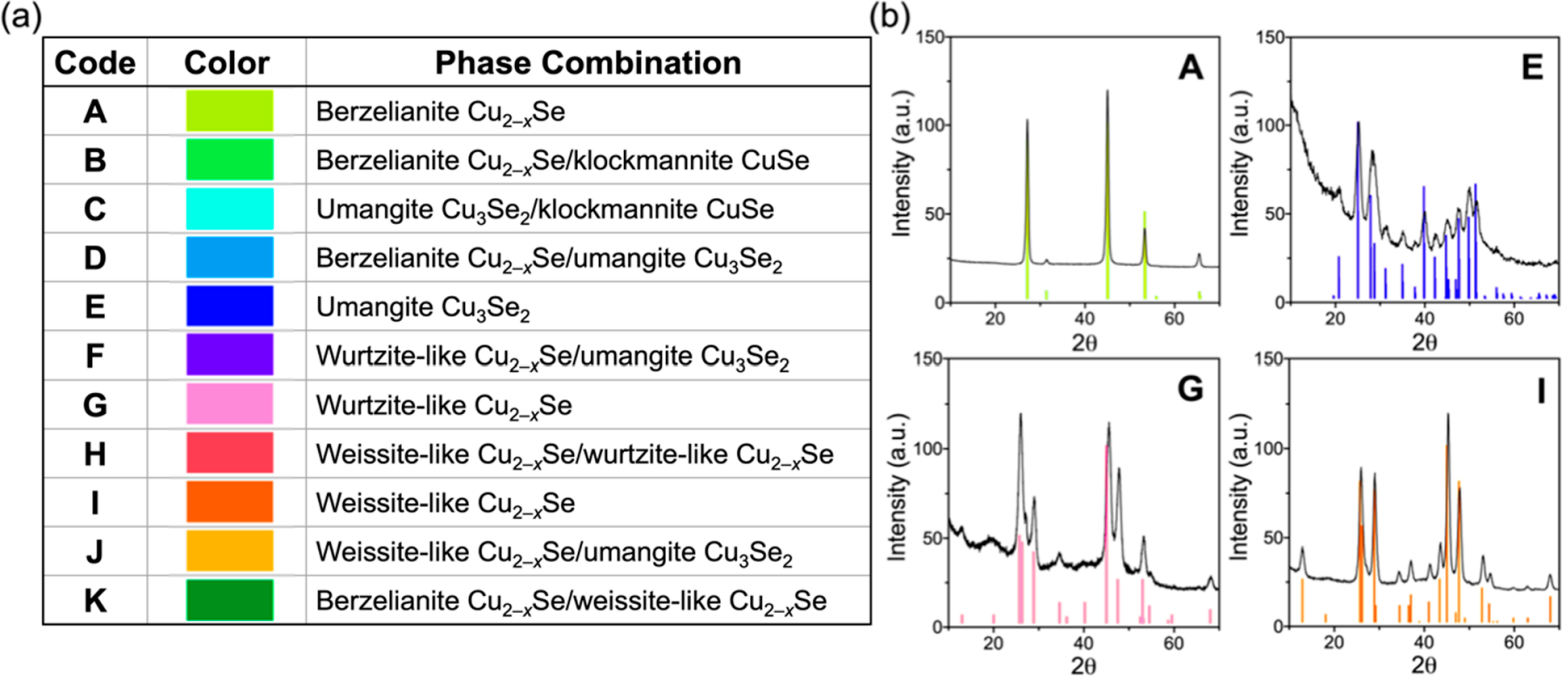
(a) Coded and color identifiers for each of the 11 unique phase combinations of copper selenide observed during the construction of the surrogate model. (b) Powder XRD patterns of four resulting phase-pure copper selenides: berzelianite Cu_2–*x*_Se (A), umangite Cu_3_Se_2_ (E), wurtzite-like Cu_2–*x*_Se (G), and weissite-like Cu_2–*x*_Se (I).

The reactions performed to construct the surrogate model were defined by (1) two full factorial screening matrices consisting of 16 experiments for each diselenide precursor, plus two center points (i.e., 34 total experiments, see Table S2), (2) two Doehlert matrices for three variables consisting of 13 experiments for each diselenide precursor (26 total experiments, see Table S3), and (3) 20 additional experiments along with replicates to assess statistical significance (see Table S4). This resulted in 80 total reactions for the surrogate model, which yielded 11 unique phase combinations of copper selenides: (A) berzelianite Cu_2–*x*_Se, (B) berzelianite Cu_2–*x*_Se/klockmannite CuSe, (C) umangite Cu_3_Se_2_/klockmannite CuSe, (D) berzelianite Cu_2–*x*_Se/umangite Cu_3_Se_2_, (E) umangite Cu_3_Se_2_, (F) wurtzite-like Cu_2–*x*_Se/umangite Cu_3_Se_2_, (G) wurtzite-like Cu_2–*x*_Se, (H) weissite-like Cu_2–*x*_Se/wurtzite-like Cu_2–*x*_Se, (I) weissite-like Cu_2–*x*_Se, (J) weissite-like Cu_2–*x*_Se/umangite Cu_3_Se_2_, and (K) berzelianite Cu_2–*x*_Se/weissite-like Cu_2–*x*_Se. For simplicity, the unique phase combinations will be referenced throughout the rest of the text by their letter and color codes, as indicated in Figure 1a, and the relative amounts of each phase present in a particular combination will not be considered at this stage of the study for experimental expediency, although others may deem quantifying phase fractions to be more practical for their particular investigations. Powder X-ray diffraction (XRD) patterns characterizing the four phase-pure copper selenides (i.e., A, E, G, I) are given in Figure 1b. The conditions for each reaction and their resulting phase combination are proved in Tables S2–S4.

### Cu–Se Phase Map by Classification Algorithm

Due to the discrete nature of categorical outcomes like phase (i.e., there is a fixed integer number of possible responses), typical regression techniques cannot be used to optimize the outcome of phase determination. This is because regression requires a continuous array of values. Classification algorithms can deal with the complexity of a categorical outcome. If prediction accuracy is statistically significant (typically, validation loss ≤0.05),^58^ then these algorithms can map the effects of several variables on a list of categorical outcomes, which, in this case, are the unique phase combinations of binary copper selenides synthesized in the study. The chosen classification algorithm was a partitioned bagged ensemble of 254 classification tree learners after Bayesian optimization of the hyperparameters. This was evaluated using leave-one-out cross-validation. The resulting model had a prediction accuracy of 95.7% with a validation loss = 0.043 and a resubstitution loss = 0.038. See the Supporting Information for further details.

**Figure 2:**
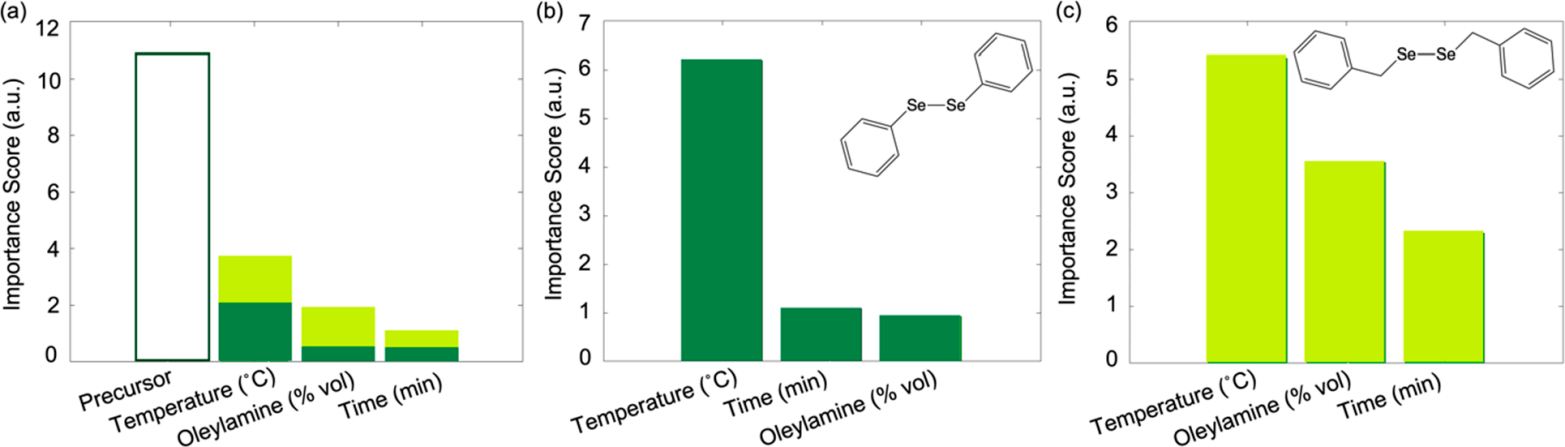
Relative importance scores of the experimental variables for (a) the entire surrogate model and separated by (b) Ph_2_Se_2_ and (c) Bn_2_Se_2_ precursors.

The effects of the experimental variables on the phase were analyzed, and importance scores were calculated for the algorithm predictions. These results indicated that C–Se bond strength was the most significant variable in determining phase, followed by temperature, volumetric ratio of oleylamine to ODE, and time, respectively (Figure 2a). Interestingly, when the data sets were separated by C–Se bond strength (or diselenide precursor), the remaining variables had different rankings of importance (Figure 2b,c). Although temperature remains the most important variable for both, the volumetric ratio of oleylamine to ODE has a significantly greater effect on reactions that use the Bn_2_Se_2_ precursor. This reinforces the notion that the mechanism of precursor conversion may differ between Bn_2_Se_2_ and Ph_2_Se_2_; for this reason, the two precursors will be assessed separately moving forward.

**Figure 3:**
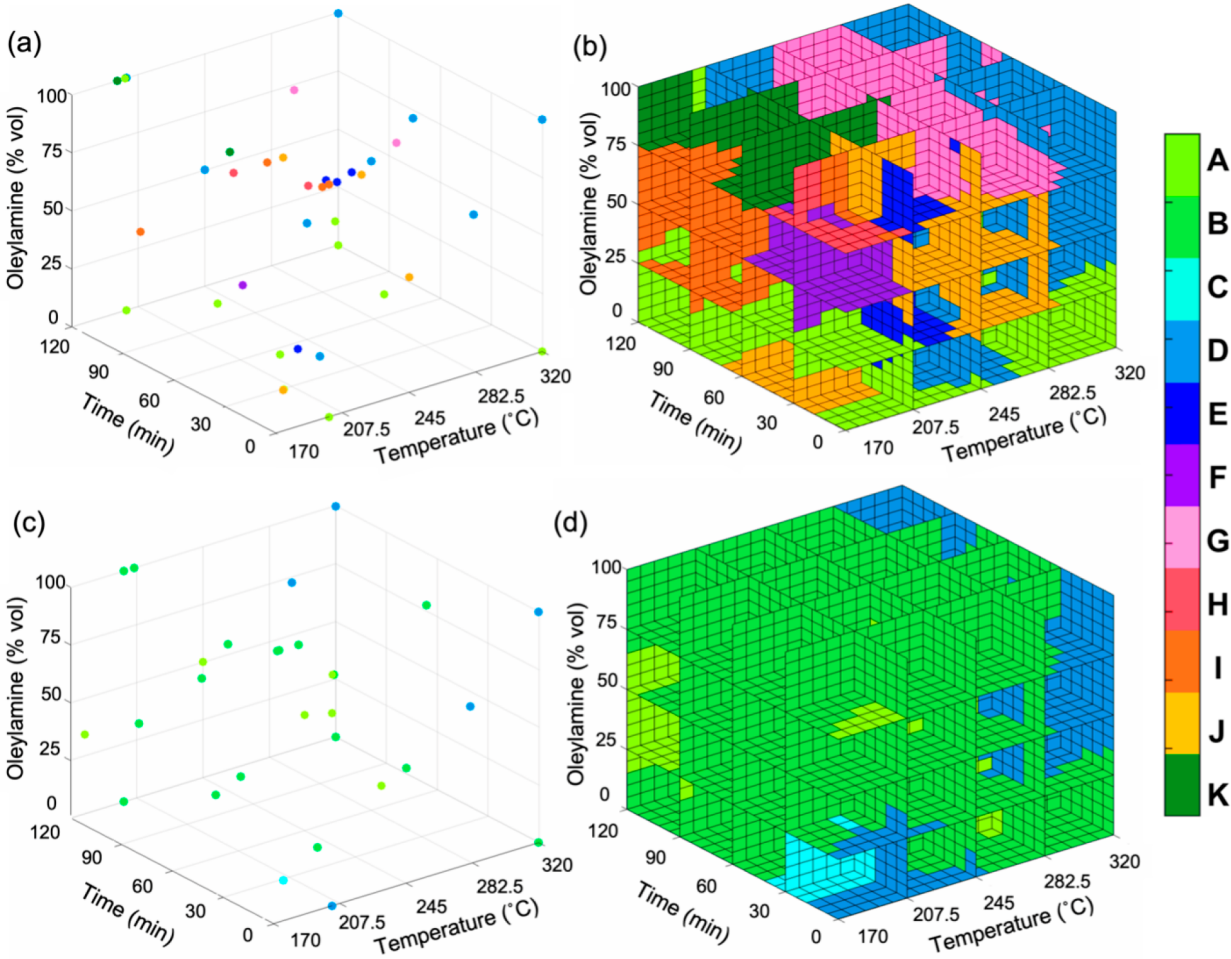
Visualization of the Cu–Se phase maps for the (a,b) Ph_2_Se_2_ and (c,d) Bn_2_Se_2_ precursors. The data points in (a) and (c) represent experiments ran in the experimental space for each respective precursor and are color coded to the phase outcome shown in the legend, where the coded letters represent the following phase combinations: (A) berzelianite Cu_2–*x*_Se, (B) berzelianite Cu_2–*x*_Se/klockmannite CuSe, (C) umangite Cu_3_Se_2_/klockmannite CuSe, (D) berzelianite Cu_2–*x*_Se/umangite Cu_3_Se_2_, (E) umangite Cu_3_Se_2_, (F) wurtzite-like Cu_2–*x*_Se/umangite Cu_3_Se_2_, (G) wurtzite-like Cu_2–*x*_Se, (H) weissite-like Cu_2–*x*_Se/wurtzite-like Cu_2–*x*_Se, (I) weissite-like Cu_2–*x*_Se, (J) weissite-like Cu_2–*x*_Se/umangite Cu_3_Se_2_, and (K) berzelianite Cu_2–*x*_Se/weissite-like Cu_2–*x*_Se. Alternative views of (b) and (d) are provided in the Supporting Information.

After analysis of the experimental results, the Ph_2_Se_2_ precursor, which has the stronger C–Se bond strength, resulted in nine distinct phase combinations of copper selenide: (A) berzelianite Cu_2–*x*_Se, (D) berzelianite Cu_2–*x*_Se/umangite Cu_3_Se_2_, (E) umangite Cu_3_Se_2_, (F) wurtzite-like Cu_2–*x*_Se/umangite Cu_3_Se_2_, (G) wurtzite-like Cu_2–*x*_Se, (H) weissite-like Cu_2–*x*_Se/wurtzite-like Cu_2–*x*_Se, (I) weissite-like Cu_2–*x*_Se, (J) weissite-like Cu_2–*x*_Se/umangite Cu_3_Se_2_, and (K) berzelianite Cu_2–*x*_Se/weissite-like Cu_2–*x*_Se (Figure 3a,b). Copper selenide phase combinations E–K were unique to the Ph_2_Se_2_ precursor. The Bn_2_Se_2_ precursor, with a weaker C–Se bond strength, resulted in four distinct phase combinations of copper selenide: (A) berzelianite Cu_2–*x*_Se, (B) berzelianite Cu_2–*x*_Se/klockmannite CuSe, (C) umangite Cu_3_Se_2_/klockmannite CuSe, and (D) berzelianite Cu_2–*x*_Se/umangite Cu_3_Se_2_ (Figure 3c,d). Phase combinations B and C were unique to the Bn_2_Se_2_ precursor, revealing that only a precursor with a lower C–Se bond strength can form the klockmannite CuSe phase, although not without the presence of a secondary phase. The only two common phases between the two precursors were berzelianite Cu_2–*x*_Se and umangite Cu_3_Se_2_; however, only the Ph_2_Se_2_ precursor rendered phase-pure umangite Cu_3_Se_2_. Similarly, only a precursor with a greater C–Se bond strength can form the metastable weissite-like and wurtzite-like Cu_2–*x*_Se phases, which were both made phase pure using the Ph_2_Se_2_ precursor. This stands in contrast to our previous synthetic explorations with these diselenide precursors, in which we were only able to isolate berzelianite Cu_2–*x*_Se and umangite Cu_3_Se_2_ phase pure.^32^

Extrapolating the reaction outcomes plotted in Figure 3a,c using a nearest neighbor likelihood algorithm yielded a prediction interpolant (i.e., a function that can be evaluated at query points) of the three-dimensional phase maps for each diselenide precursor, as illustrated in Figures 3b,d and further described in the Supporting Information. Looking specifically at the Ph_2_Se_2_ precursor, the stronger C–Se bond strength leads to a higher energy barrier for precursor conversion. Usually, isolation of metastable materials occurs when the kinetics are rapid enough that the reaction coordinate cannot reach equilibrium and becomes trapped in a local free energy minimum. Therefore, it seems counterintuitive that the precursor with a higher barrier and slower kinetics to conversion leads to kinetic trapping. Despite this, the Ph_2_Se_2_ precursor facilitates a much richer Cu–Se phase space, which is exemplified by the nine phase combinations achieved using this precursor versus the four phase combinations achieved with the Bn_2_Se_2_ precursor. This suggests that the various metastable copper selenides themselves may have a high activation barrier to conversion to the more thermodynamically stable copper selenides and that the Ph_2_Se_2_ precursor has a distinctive decomposition mechanism that leads to these metastable species, rather than just a difference in activation barrier.^42^

**Figure 4:**
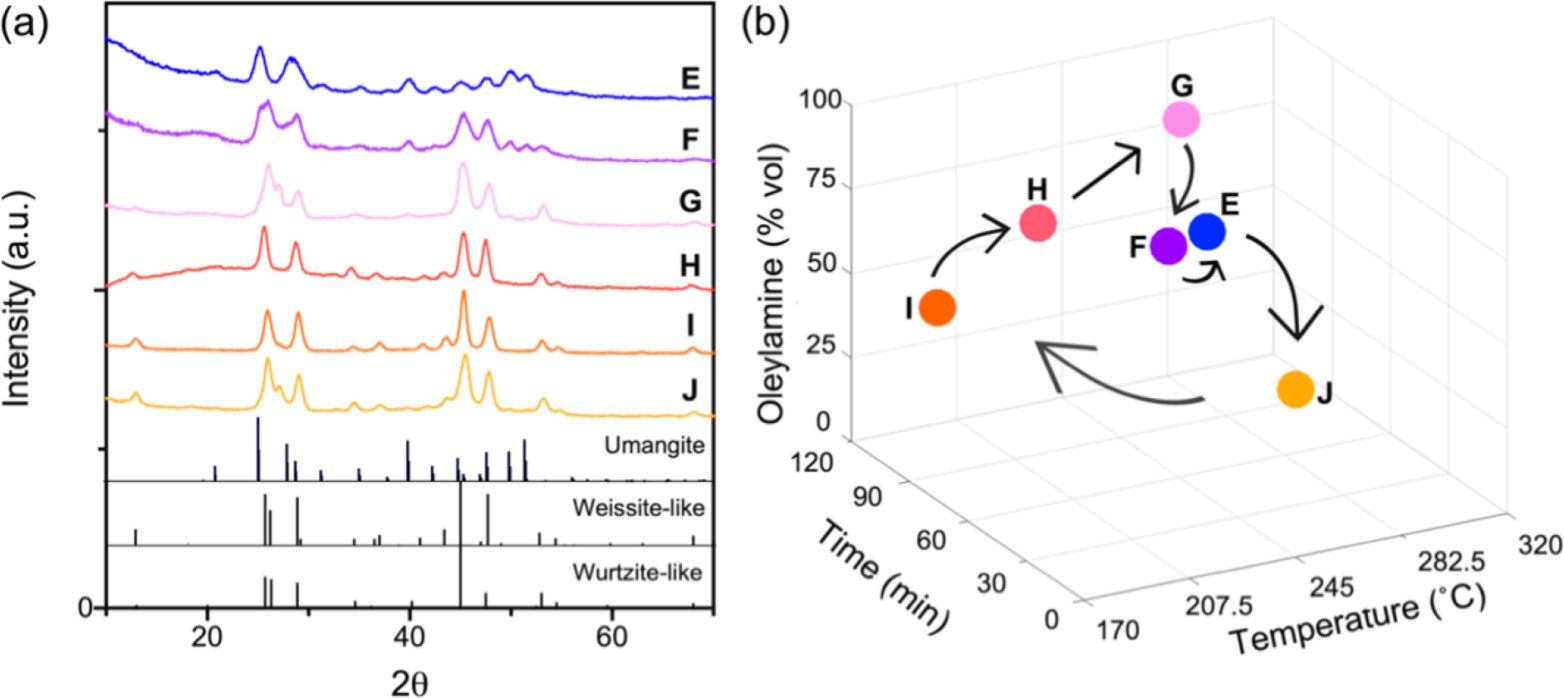
(a) Powder XRD patterns of phase combinations that result in the (b) subregion of the phase map that is bound by the area of lower temperatures and higher volumetric ratios of oleylamine with the Ph_2_Se_2_ precursor. The coded letters represent the following phase combinations: (E) umangite Cu_3_Se_2_, (F) wurtzite-like Cu_2–*x*_Se/umangite Cu_3_Se_2_, (G) wurtzite-like Cu_2–*x*_Se, (H) weissite-like Cu_2–*x*_Se/wurtzite-like Cu_2–*x*_Se, (I) weissite-like Cu_2–*x*_Se, and (J) weissite-like Cu_2–*x*_Se/umangite Cu_3_Se_2_.

Berzelianite Cu_2–*x*_Se forms under oleylamine-poor reaction conditions when using Ph_2_Se_2_, whereas umangite Cu_3_Se_2_ forms under more oleylamine-rich reaction conditions (Figure 3b). This is counterintuitive since the more reducing conditions introduced with greater volumetric ratios of oleylamine result in Cu_3_Se_2_, which formally contains Cu^2+^,^61^ although we should note that other studies have assigned copper as being monovalent with the oxidation state of selenium being −3/2.^62^ Nonetheless, this highlights the value of using such phase maps and the information they can provide. The phase map in Figure 3b pinpoints the locations of the metastable weissite-like and wurtzite-like Cu_2–*x*_Se phases, indicating regions that border between thermodynamic and kinetic stability. These regions are bound by the areas of lower temperatures and higher volumetric ratios of oleylamine. The change in conditions that separate three phases, umangite Cu_3_Se_2_, weissite-like Cu_2–*x*_Se, and wurtzite-like Cu_2–*x*_Se (E, G, and I, respectively), can be seen in greater detail in the subregion of the experimental space provided in Figure 4. The wurtzite-like polymorph forms at higher temperatures and high volumetric ratios of oleylamine, which transitions into the umangite Cu_3_Se_2_ phase within a small window of slightly lower temperatures and shorter reaction times. The phase map eventually transitions into the weissite-like Cu_2–*x*_Se phase at longer reaction times, with a very small temperature difference of 2–3 °C separating umangite Cu_3_Se_2_ and wurtzite-like Cu_2–*x*_Se, which is very challenging to pinpoint using traditional, Edisonian methods. These results are consistent with umangite Cu_3_Se_2_ being a low-temperature phase on the thermodynamic phase diagram;^21^ however, both weissite-like and wurtzite-like Cu_2–*x*_Se are metastable phases that are not present on the thermodynamic phase diagram and had not been observed in any of our previous exploratory chemistry with these two diselenide precursors. This further illustrates the power of this approach; that is, we were able to isolate two phase-pure metastable materials within a complex experimental space, where a reaction temperature difference of only a few degrees can separate them from umangite Cu_3_Se_2_.

The fact that these three phases (i.e., umangite Cu_3_Se_2_ (E), weissite-like Cu_2–*x*_Se (I), and wurtzite-like Cu_2–*x*_Se (G)) all exist within this subregion of the experimental space is perhaps not surprising given the fact that both umangite Cu_3_Se_2_ and weissite-like Cu_2–*x*_Se can be viewed as slight distortions of the wurtzite-like anionic sublattice.^28,63^ The anion sublattice of the umangite Cu_3_Se_2_ structure maintains a quasi-planar hexagonal framework of Se^2–^ that stacks in an alternating ABAB fashion. The interplanar distance between these anion layers is 3.2 Å, which is similar to the anisotropic hexagonal wurtzite-like structure with the hexagonally close-packed layers stacked along the *c*-axis with a *d*-spacing of 3.4 Å.^29,30^ Similarly, in the weissite-like structure, Cu^+^ occupies trigonal and tetrahedral sites in a slightly distorted hexagonal Se^2–^ sublattice with a *d*-spacing of 3.4 Å.^28^ This structure has Cu-rich and Cu-deficient layers sandwiched in a distorted hexagonal layer of Se^2–^.

**Figure 5:**
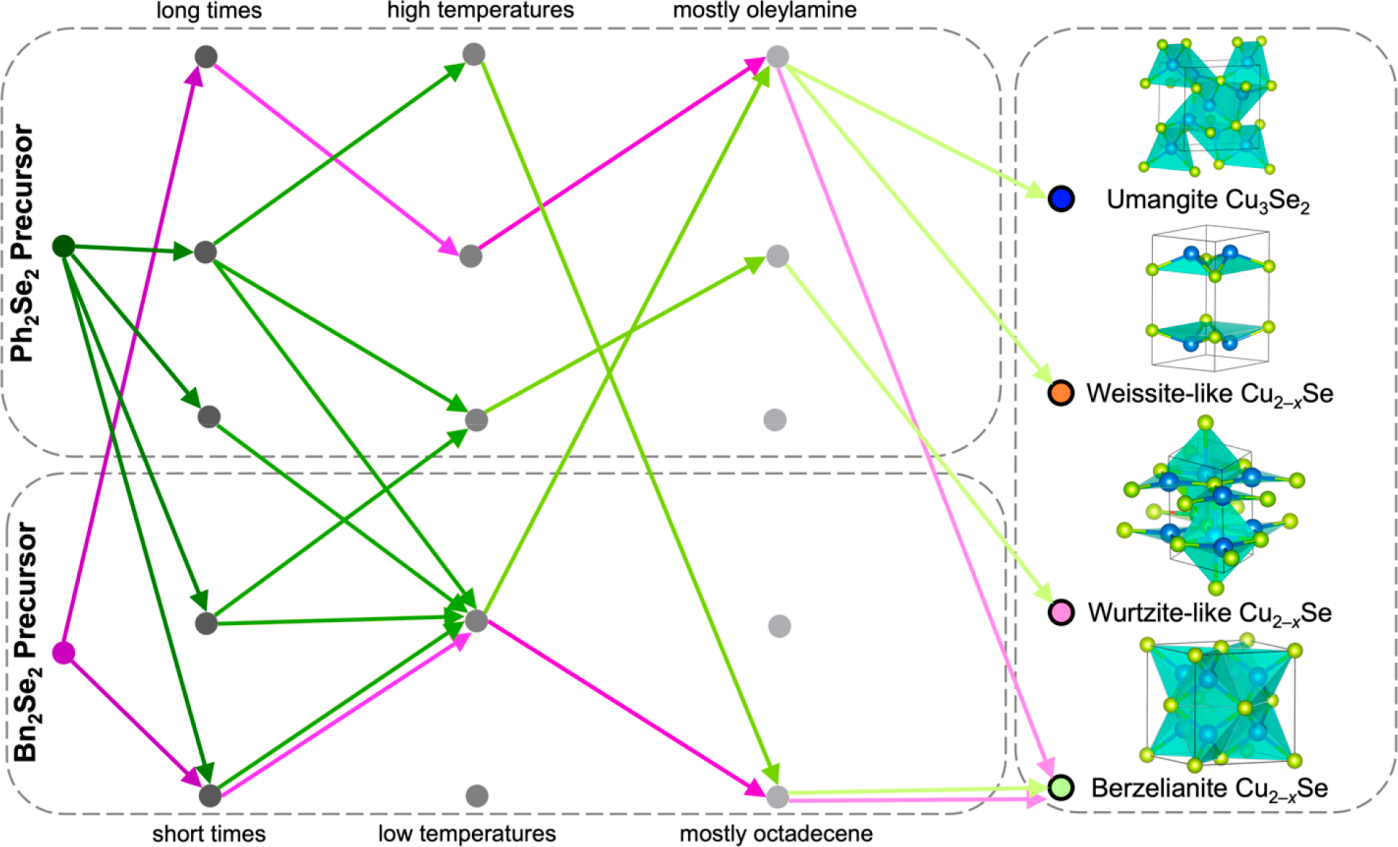
Simplified decision tree for the Cu–Se phase map predicted by the classification algorithm, with Bn_2_Se_2_ precursor pathways to phase-pure products indicated by purple arrows and Ph_2_Se_2_ precursor pathways to phase-pure products indicated by green arrows.

For the Bn_2_Se_2_ precursor, most of the experimental space returns mixtures of phases (Figure 3c,d). As opposed to Ph_2_Se_2_, phase-pure berzelianite Cu_2–*x*_Se forms under more oleylamine-rich reaction conditions with Bn_2_Se_2_, illustrating that the relationship between the diselenide precursor and oleylamine is countercorrelated between the two precursors. While most of this experimental space returns phase combinations with berzelianite Cu_2–*x*_Se, the Bn_2_Se_2_ precursor yields phase combinations containing klockmannite CuSe, whereas Ph_2_Se_2_ does not. Klockmannite CuSe possesses some degree of Se–Se bonding in the structure.^64^ Klockmannite CuSe may be observed with Bn_2_Se_2_ because its Se–Se bond (BDE = 53 kcal mol^–1^) is 11 kcal mol^–1^ stronger than the Se–Se bond in Ph_2_Se_2_; therefore, some degree of precursor conversion may result in Se_2_^2–^. A simplified decision tree of the routes to synthesize phase-pure copper selenides from this stage of the study is illustrated in Figure 5. The full classification trees, showing more specific experimental values and the prescriptive synthetic routes to mixed phase combinations, can be found in Figures S8 and S9 in the Supporting Information.

### Predictive Phase Determination of Klockmannite CuSe *via* Classification Model

To illustrate the power of classification as a tool for predictive phase determination, we next addressed our inability to isolate klockmannite CuSe within the bounds of the experimental space. Although klockmannite is observed in the Bn_2_Se_2_ portion of the phase map, it is only found in combination with berzelianite Cu_2–*x*_Se or umangite Cu_3_Se_2_. Thus, the model was used to predict a synthetic route to klockmannite CuSe using the Bn_2_Se_2_ precursor. Like the bounded region of the metastable phases discussed for Ph_2_Se_2_ (*vide supra*), the target phase was predicted to lie somewhere in the region near the boundary of klockmannite combined with umangite Cu_3_Se_2_ (C) and klockmannite combined with berzelianite Cu_2–*x*_Se (B).

**Figure 6:**
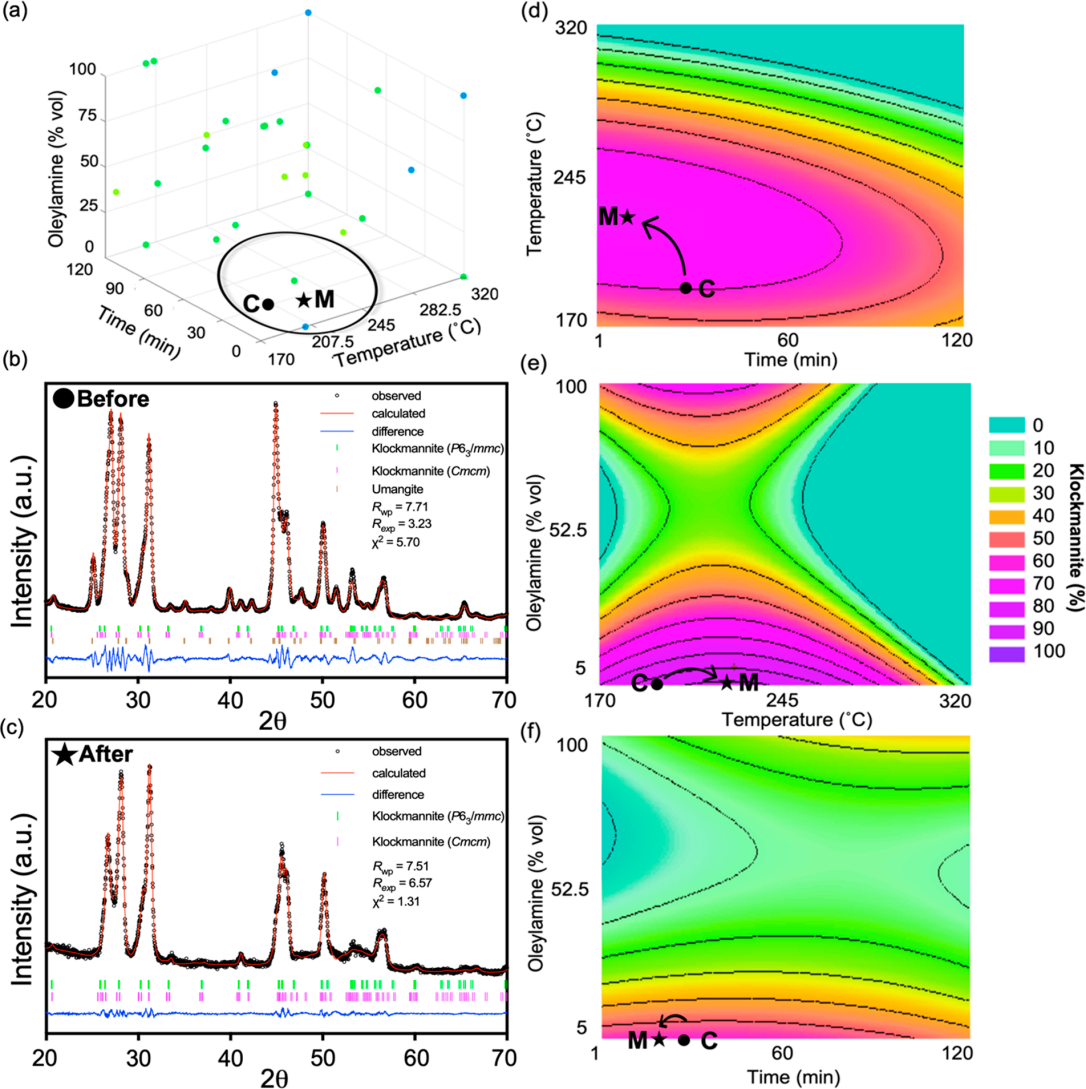
(a) Cu–Se phase map for the Bn_2_Se_2_ precursor, with the subregion of interest circled in black. The initial experimental conditions are identified by a black circle (C) and the target conditions for synthesizing klockmannite CuSe are indicated by a star (M). (b) Rietveld refinement of the XRD pattern collected on the mixture of klockmannite CuSe and umangite Cu_3_Se_2_ (C) before phase targeting. (c) Rietveld refinement of the XRD pattern collected on the klockmannite CuSe (M) after phase targeting (λ = 1.54 Å). (d–f) Response surfaces giving the predicted relative phase purity of klockmannite CuSe throughout the experimental space, with the initial experimental conditions and target conditions shown by the black circle (C) and star (M), respectively.

This subregion of the experimental space is characterized by short to medium reaction times, low temperatures, and lower volumetric ratios of oleylamine to ODE, as indicated by the area circled in Figure 6a. The classification model predicted that this region of klockmannite CuSe (coded M) can be specifically defined by the following experimental conditions: 203.5–234.3 °C, <9.3 vol% of oleylamine in ODE, and 22.5–30.4 min. This is illustrated in Figure S9 by following the predicted routes that lie between phase combinations B and C, which are the only two-phase combinations that include klockmannite CuSe. These classification tree pathways reflect the interdependencies between of the parameters in reaching a particular categorical product. For klockmannite CuSe, the path indicates that the volume fraction of oleylamine is dependent on temperature (Figure S9). Interestingly, this volume fraction of oleylamine to ODE falls in the undefined region of the simplified decision tree, as seen in Figure 5. Using these synthetic guidelines, three reactions were conducted at 205, 215, and 225 °C with aliquots taken at 1, 15, 30, 45, 60, and 120 min to better sample the phase outcome over time in this region, since time was predicted to be the least significant variable, and pin-pointing the experimental conditions to synthesize a previously unisolable phase predicted to only exist in a very small region of the parameter space is difficult. Because high volumetric ratios of oleylamine to ODE were projected to hinder the isolation of CuSe, it was kept at a lower value (5 vol%).

The relative amounts of klockmannite CuSe synthesized in each reaction were estimated from diffraction peak intensities (of the 100% intensity peak), with the reaction at 205 °C containing ca. 40% klockmannite CuSe and 60% berzelianite Cu_2–*x*_Se, the reaction at 215 °C containing ca. 50% klockmannite and 50% berzelianite, and the reaction at 225 °C containing ca. 80% klockmannite and 20% berzelianite (Figure S10). The very crude, yet easy, estimation of phase fractions by quantification of diffraction intensities was found to be sufficient for this optimization and enabled an analysis of variable significance *via* ANOVA, as shown in the Pareto chart in Figure S13a. This analysis indicated that the quadratic interaction of temperature with itself had the most significant effect on klockmannite CuSe formation, followed by the linear effect of low vol% of oleylamine, and its quadratic interaction with itself. This is in good agreement with the pathway indicated by the classification tree in Figure S9. The model predictions were experimentally validated by these initial aliquots, which showed maximum fractions of klockmannite between 15 and 30 min for all three temperatures. After 45 min, the fraction of klockmannite CuSe plateaued at the lower two temperatures and the 225 °C reaction began to produce umangite Cu_3_Se_2_. The mixture of klockmannite CuSe, umangite Cu_3_Se_2_, and berzelianite Cu_2–*x*_Se from the reaction at 225 °C represents a twelfth unique phase combination (coded L). This insight prompted two more reactions at 220 and 230 °C, where aliquots were taken at 1, 5, 10, 15, 20, and 25 min. These reactions resulted in ca. 70% klockmannite and 60% klockmannite, respectively (Figure S11). Reaction times ranging between 5 and 25 min seemed to have negligible effects on the phase combination in this temperature range, further validating the model prediction that time was the least important variable in determining phase for this system.

These data were combined with the surrogate model experiments that yielded phase combinations that included klockmannite CuSe. Response surface methodology was used to fit the data to a polynomial model, resulting in the response surfaces shown in Figure 6d–f. These additional data were similarly added into the trained classifier, which maintained its prediction accuracy across several train/test splits, affording further validation of model accuracy (see Supporting Information). After extrapolating the data, the optimal reaction conditions were predicted to be a reaction time of 24.3 min, a reaction temperature of 223.5 °C, and 4.7 vol% oleylamine in ODE, corroborating the initial prediction made by the classification model. These synthetic conditions (predicted *via* RSM) were run in triplicate to validate the model, which, in each case, identically resulted in klockmannite CuSe. This represents the 13th unique phase or phase combination in this experimental space (coded M). A Rietveld refinement of the XRD pattern is given in Figure 6c, with χ^2^ = 1.31. We compare this to a Rietveld refinement of the XRD pattern with the highest fraction of klockmannite CuSe from the original surrogate model data (i.e., 203.5 °C, 30 min, and 5 vol% oleylamine in ODE), which had a χ^2^ = 5.70 and shows a combination of umangite Cu_3_Se_2_ and klockmannite (C) (Figure 6b). These results demonstrate how the classification algorithm allowed us to isolate klockmannite CuSe in only six additional experiments, past the construction of the initial surrogate model.

A subsequent aliquot study at 223.5 °C (i.e., the predicted optimal temperature) was conducted to assess if the predicted time of 24.3 min was significant, considering that time had seemed to have negligible effects on phase at a temperature only 3.5 °C lower in the study at 220 °C. The results showed slight impurities at both 20 and 30 min (Figure S12), indicating that klockmannite CuSe occupies an extremely small region of the phase map and requires a very precise set of reaction conditions for its isolation. The small change in experimental conditions that shifts the synthetic outcome from a two-phase mixture to klockmannite CuSe further highlights the utility of this approach to target a desired phase that was previously inaccessible in a small subregion of a large, high-dimensional experimental space.

## Conclusions

The large experimental space used to synthesize binary copper selenides was mapped for four variables: C–Se precursor bond strength (Ph_2_Se_2_ or Bn_2_Se_2_), volumetric ratio of oleylamine to ODE, reaction time, and temperature. Patterns in the data were analyzed by using a data-driven classification algorithm. After the experiments were performed dictated by orthogonal screening and optimization design matrices, a surrogate model was created to provide experimental data for the training and testing of a classification model. Calculation of variable importance scores and multivariate, high-dimensional phase maps created from the resulting classification tree and likelihood algorithms enabled detailed conclusions to be drawn about the relationships between experimental variables and phase. The type of diselenide precursor was shown to be the most important factor for phase determination, followed by temperature, with certain phases lying in very narrow temperature ranges within the phase map. The importance of the volumetric ratio of oleylamine to ODE and time depended on the precursor type, suggesting that the resulting phase is dictated by different precursor conversion mechanisms. The precursor with a higher C–Se bond strength (Ph_2_Se_2_) led to a richer phase map with more unique phase combinations, allowing the isolation of three distinct phases, including two metastable phases.

The phase maps and insights from data-driven classification acted as a guide for the accelerated isolation of klockmannite CuSe in just six additional experiments. The isolation of this phase is significant because of the presence of Se–Se bonding within this structure, making it distinct from the other isolated phases, and it is accessed by the Bn_2_Se_2_ precursor with the stronger Se–Se bond. This is the first example of high-dimensional mapping of a multiphase, multivariate domain with mixed categorical and discrete variables and the first example of data-driven classification techniques being employed to target a previously inaccessible phase within an experimental space. The resulting phase maps not only streamline phase determination in the complex binary Cu–Se system studied here but will be broadly applicable to the targeted *chimie douce* synthesis of other materials classes as well. The ability of these phase maps to capture both thermodynamic and kinetic complexity in a way that typical phase diagrams do not is incredibly valuable for experimentalists in trying to isolate new materials. Considering that there are many other material properties that depend on a combination of both kinetic and thermodynamic factors (e.g., material size and dimensionality, morphology, and composition),^11,18,20,48^ such information-rich phase maps will have value across a wide array of problems.

Furthermore, although the focus of this study was strictly phase-driven, successful results stemming from estimation of the relative percentages of klockmannite CuSe phase in each reaction act as a proof of concept for phase analysis as a continuous variable *via* much less time intensive techniques to quantify phase, which would typically require *n* number of XRD refinements for a data set composed of *n* experiments. This opens the door for facile regression-based phase investigations that also include morphological characterization and control as additional responses as an extension to colloidal nanocrystals. This is particularly beneficial for materials whose performance in applications is dependent on morphology (e.g., nanocrystalline materials for catalysis) and generalizes the possibilities of extending this technique to a vast number of materials systems.

## Experimental Procedures

### Materials and General Procedures

Copper(II) dichloride dihydrate (CuCl_2_·2H_2_O, 99%, Sigma-Aldrich), sodium oleate (>97%, TCI America), diphenyl diselenide (Ph_2_Se_2_, 98%, Sigma-Aldrich), dibenzyl diselenide (Bn_2_Se_2_, 98%, Alfa Aesar), 1-octadecence (90%, Sigma-Aldrich), and oleylamine (70%, Sigma-Aldrich) were obtained as indicated. Oleylamine and 1-octadecene were degassed under a vacuum at 120 °C for 4 h and then overnight at room temperature prior to use. All reactions were conducted under a flowing nitrogen atmosphere using standard Schlenk techniques. All reactions employed J-KEM temperature controllers with in situ thermocouples to control and monitor the temperature of the reaction vessel.

### Synthesis of Cu(oleate)_2_

An adapted literature approach was used.^32^ Sodium oleate (3.0 g, 9.9 mmol) and CuCl_2_·2H_2_O (0.84 g, 4.9 mmol) were placed in a round-bottom flask. A solution containing 10 mL of ethanol, 8 mL of DI water, and 17 mL of hexanes was added to the flask, and the reaction mixture was heated to 70 °C. After 25 min, an additional 10 mL of hexanes were added to the solution, and the flask was kept at 70 °C for 4 h. After cooling, the product was collected in the hexanes layer, separated, and washed three times with 30 mL of DI water in a separatory funnel. The hexanes layer was collected, and all volatiles were removed to produce a blue-green Cu(oleate)_2_ product.

### Synthesis of Copper Selenide

Cu(oleate)_2_ (0.16 g, 0.25 mmol) and R_2_Se_2_ (0.25 mmol, 0.0850 g of R = Bn, or 0.0785 g of R = Ph) were placed in a three-neck round-bottom flask and dissolved in 12 mL of varying volumetric ratios of oleylamine and ODE under flowing nitrogen. The flask was then heated to 70 °C and degassed for 30 min under vacuum. The temperature was raised to 140 °C, and the flask was degassed for an additional 30 min. At this point, the solution is clear and has a teal color. The reaction temperature was ramped to the indicated set point under flowing nitrogen at 5–6 °C·min^–1^ and held at that temperature for the duration of the reaction. The reaction solution was then thermally quenched by quickly removing it from the heating mantle and immediately placing it in a room-temperature water bath. Hexanes (<5 mL) were added to the reaction suspension, which was then removed from the round-bottom flask and split equally between two 50 mL centrifuge tubes that were filled to 40 mL with ethanol, sonicated for 10 min, and centrifuged at 6000 rpm for 4.5 min. This washing procedure was repeated twice with 5 mL of hexanes and 40 mL of ethanol. After the final centrifugation step, the copper selenide was isolated and dried under flowing nitrogen at room temperature to give a powder for X-ray diffraction.

### Characterization

Powder X-ray diffraction (XRD) measurements were collected from 10° to 70° 2θ with a step size of 1° min^–1^ on a Rigaku Ultima IV powder X-ray diffractometer using Cu Kα radiation (λ = 1.54 Å). Powder samples were prepared on a zero-diffraction silicon substrate. Rietveld structural refinements were performed using the BGMN/Profex 5.2.0 software.^65,66^ ICSD structural files of each aforementioned Cu–Se phase were included in the fitting to determine the phases present. The following parameters were refined: (1) scale factor, (2) background, (3) peak shape, and peak broadening lead by crystallite size and microstrain, (4) lattice parameters, (5) fractional atomic coordinates of atoms when allowed by symmetry and microstrain effects, (6) peak intensities from preferred orientation effects, and (7) an isotropic thermal parameter for each crystallographic site in the crystal structures. The *R*_wp_ and χ^2^ were employed to assess the quality of the refined structural models.

## Supporting Information Available

Figures and tables detailing all reactions that comprise the surrogate model and their resultant phase combination; explanation of the algorithms and validation techniques used in training and testing; full decision trees output from the trained classifier; additional XRD patterns and figures illustrating statistical analysis from the optimization of phase pure klockmannite CuSe phase (PDF)
