# Supplementary material for: Predictive Synthesis of Copper Selenides Using a Multidimensional Phase Map Constructed with a Data-Driven Classifier
Source: J Am Chem Soc. 2023 Aug 4;145(32):17954–64. doi: 10.1021/jacs.3c05490 (PMC10436277; doi:10.1021/jacs.3c05490)
Supplement: Supplementary file 1 — ja3c05490_si_001.pdf [file ja3c05490_si_001.pdf]

SUPPORTING INFORMATION

Predictive Synthesis of Copper Selenides using a Multidimensional Phase Map Constructed with a Data-Driven Classifier

Emily M. Williamson, Zhaohong Sun, Bryce A. Tappan, and Richard L. Brutchey*

Department of Chemistry, University of Southern California, Los Angeles, CA, 90089, USA

*Email: brutchey@usc.edu

Table of Contents

1. Phases of Binary Cu–Se........ S2
2. Surrogate Model........ S3
3. Classification........ S8
4. Isolating the Target Klockmannite CuSe Phase........ S13
5. References........ S16

1. Phases of Binary Cu–Se

Table S1. Phases of Binary Copper Selenide and Corresponding Crystallographic Information[1–6]

Phase		Structure	Space Group	Select XRD Peaks (2θ)[a]	Temperature Range on Bulk Phase Diagram
berzelianite α-$Cu_{2-x}Se$		Anti-fluorite (cubic)	$Fm\bar{3}m$	**44**, 27, 52	< 105 °C
weissite-like $Cu_{2-x}Se$		Trigonal	$P\bar{3}m1$	**45, 48**, 26, 29	metastable
wurtzite-like $Cu_{2-x}Se$		Hexagonal	$P6_3mc$	**45, 26,** 48, 29	metastable
umangite Cu_3Se_2		Tetragonal	$P\bar{4}2_1m$	**25, 50**, 52, 40	< 113 °C
klockmannite	β-CuSe	Orthorhombic	$Cmcm$	**45, 28, 31**	54-137 °C
	γ-CuSe	Hexagonal	$P6_3/mmc$	**28, 46,** 31, 50, 56	137-380 °C
marcasitic krutaite $CuSe_2$		Orthorhombic	$Pnnm$	**29, 33**, 34, 46	< 332 °C, low pressure phase
pyritic krutaite $CuSe_2$		Cubic	$Pa\bar{3}$	**33, 36**, 29, 49	< 332 °C, high pressure phase

[a] The two highest intensity XRD peaks are bolded.

2. Surrogate Model

Table S2. Full Factorial Screening Reactions and Responses for the Surrogate Model

Rxn.	Precursor	Time	Temp.	OAm/ODE	Time	Temp.	OAm/ ODE	Phase
		coded	coded	coded	min	˚C	%vol	
1	Ph_2Se_2	-1	-0.6	1	1	200	100	wurtzite-like $Cu_{2-x}Se$/umangite Cu_3Se_2
2	Ph_2Se_2	-1	-0.6	-1	1	200	5	berzelianite $Cu_{2-x}Se$
3	Bn_2Se_2	-1	-0.56	-1	1	202.9	5	berzelianite $Cu_{2-x}Se$/umangite Cu_3Se_2
4	Bn_2Se_2	-0.513	-0.6	1	30	200	100	berzelianite $Cu_{2-x}Se$/klockmannite CuSe
5	Ph_2Se_2	-0.513	-0.573	-1	30	202	5	weissite-like $Cu_{2-x}Se$/umangite Cu_3Se_2
6	Bn_2Se_2	-1	-0.556	1	1	203.3	100	berzelianite $Cu_{2-x}Se$
7	Ph_2Se_2	1	1	1	120	320	100	berzelianite $Cu_{2-x}Se$/umangite Cu_3Se_2
8	Bn_2Se_2	1	1	1	120	320	100	berzelianite $Cu_{2-x}Se$/umangite Cu_3Se_2
9	Ph_2Se_2	1	-0.59	1	120	200.7	100	berzelianite $Cu_{2-x}Se$/umangite Cu_3Se_2
10	Ph_2Se_2	-1	1	1	1	320	100	berzelianite $Cu_{2-x}Se$/umangite Cu_3Se_2
11	Ph_2Se_2	-1	1	-1	1	320	5	berzelianite $Cu_{2-x}Se$
12	Ph_2Se_2	1	-0.59	-1	120	200.6	5	berzelianite $Cu_{2-x}Se$
13	Ph_2Se_2	1	1	-1	120	320	5	berzelianite $Cu_{2-x}Se$
14	Bn_2Se_2	-1	1	1	1	320	100	berzelianite $Cu_{2-x}Se$/umangite Cu_3Se_2
15	Bn_2Se_2	-1	1	-1	1	320	5	berzelianite $Cu_{2-x}Se$/klockmannite CuSe
16	Bn_2Se_2	1	-0.6	-1	120	200	5	berzelianite $Cu_{2-x}Se$/klockmannite CuSe
17	Bn_2Se_2	1	1	-1	120	320	5	berzelianite $Cu_{2-x}Se$/klockmannite CuSe
18	Bn_2Se_2	1	-0.6	1	120	200	100	berzelianite $Cu_{2-x}Se$/klockmannite CuSe
19	Bn_2Se_2	-0.008	0.2	-0.053	60	260	50	berzelianite $Cu_{2-x}Se$
20	Ph_2Se_2	-0.008	0.205	-0.053	60	260	50	berzelianite $Cu_{2-x}Se$
21	Ph_2Se_2	-0.513	-0.947	1	30	174	100	weissite $Cu_{2-x}Se$/wurtzite $Cu_{2-x}Se$
22	Ph_2Se_2	-0.513	0.4	1	30	275	100	berzelianite $Cu_{2-x}Se$/umangite Cu_3Se_2
23	Ph_2Se_2	-0.513	-0.567	-1	30	202.5	5	berzelianite $Cu_{2-x}Se$
24	Ph_2Se_2	-0.933	-0.707	1	5	192	100	weissite $Cu_{2-x}Se$/wurtzite $Cu_{2-x}Se$
25	Ph_2Se_2	-0.513	-0.691	1	30	193.2	100	weissite $Cu_{2-x}Se$
26	Ph_2Se_2	-0.513	-0.597	-0.684	30	200.2	20	berzelianite $Cu_{2-x}Se$
27	Bn_2Se_2	-0.513	-0.588	1	30	200.9	100	berzelianite $Cu_{2-x}Se$/klockmannite CuSe
28	Bn_2Se_2	1	-0.519	1	120	206.1	100	berzelianite $Cu_{2-x}Se$/klockmannite CuSe
29	Ph_2Se_2	1	-0.597	1	120	200.2	100	berzelianite $Cu_{2-x}Se$
30	Bn_2Se_2	-1	-0.548	1	1	203.9	100	berzelianite $Cu_{2-x}Se$/klockmannite CuSe
31	Bn_2Se_2	-0.513	0.525	1	30	284.4	100	berzelianite $Cu_{2-x}Se$/klockmannite CuSe
32	Bn_2Se_2	-0.513	-0.441	1	30	211.9	100	berzelianite $Cu_{2-x}Se$/klockmannite CuSe
33	Bn_2Se_2	-0.513	-0.553	-1	30	203.5	5	klockmannite CuSe/umangite Cu_3Se_2
34	Ph_2Se_2	-1	-0.653	1	1	196	100	weissite $Cu_{2-x}Se$

Table S3. Full Doehlert Optimization Design and Responses for the Surrogate Model

Rxn.	Precursor	Time	Temp.	OAm/ODE	Time	Temp.	OAm/ODE	Phase
		coded	coded	coded	min	˚C	%vol	
1	Ph_2Se_2	-1	0	0	1	245	52.2	weissite $Cu_{2-x}Se$/umangite Cu_3Se_2
2	Bn_2Se_2	-1	0	0	1	245	52.5	berzelianite $Cu_{2-x}Se$/klockmannite CuSe
3	Bn_2Se_2	1	0	0	120	245	52.5	berzelianite $Cu_{2-x}Se$
4	Ph_2Se_2	1	0	0	120	245	52.5	berzelianite $Cu_{2-x}Se$/umangite Cu_3Se_2
5	Ph_2Se_2	0	0	0	60.5	245	52.5	berzelianite $Cu_{2-x}Se$/umangite Cu_3Se_2
6	Bn_2Se_2	0	0	0	60.5	245	52.5	berzelianite $Cu_{2-x}Se$
7	Ph_2Se_2	-0.5	-0.866	0	30.75	180.1	52.5	berzelianite $Cu_{2-x}Se$
8	Ph_2Se_2	0.5	0.866	0	90.25	309.9	52.5	berzelianite $Cu_{2-x}Se$/umangite Cu_3Se_2
9	Bn_2Se_2	0.5	0.289	0.817	90.25	266.7	91.3	berzelianite $Cu_{2-x}Se$
10	Ph_2Se_2	0.5	0.289	0.817	90.25	266.7	91.3	wurtzite $Cu_{2-x}Se$
11	Ph_2Se_2	-0.5	-0.866	0	30.75	180.1	52.5	wurtzite $Cu_{2-x}Se$/umangite Cu_3Se_2
12	Ph_2Se_2	-0.5	-0.289	-0.817	30.75	223.3	13.7	berzelianite $Cu_{2-x}Se$/umangite Cu_3Se_2
13	Ph_2Se_2	0.5	-0.866	0	90.25	180.1	52.5	weissite $Cu_{2-x}Se$
14	Ph_2Se_2	0.5	-0.289	-0.817	90.25	223.3	13.7	berzelianite $Cu_{2-x}Se$
15	Ph_2Se_2	-0.5	0.866	0	30.75	309.9	52.5	berzelianite $Cu_{2-x}Se$ /umangite Cu_3Se_2
16	Ph_2Se_2	0	0.577	-0.817	60.5	288.3	13.7	berzelianite $Cu_{2-x}Se$
17	Ph_2Se_2	0.5	0.289	0.817	90.25	266.7	91.3	wurtzite $Cu_{2-x}Se$
18	Ph_2Se_2	0	-0.577	0.817	60.5	201.7	91.3	weissite $Cu_{2-x}Se$/berzelianite $Cu_{2-x}Se$
19	Bn_2Se_2	-0.5	-0.866	0	30.75	180.1	52.5	berzelianite $Cu_{2-x}Se$/klockmannite CuSe
20	Bn_2Se_2	-0.5	-0.289	-0.817	30.75	223.3	13.7	berzelianite $Cu_{2-x}Se$/klockmannite CuSe
21	Bn_2Se_2	0.5	-0.866	0	90.25	180.1	52.5	berzelianite $Cu_{2-x}Se$/klockmannite CuSe
22	Bn_2Se_2	0.5	-0.289	-0.817	90.25	223.3	13.7	berzelianite $Cu_{2-x}Se$/klockmannite CuSe
23	Bn_2Se_2	-0.5	0.866	0	30.75	309.9	52.5	berzelianite $Cu_{2-x}Se$/umangite Cu_3Se_2
24	Bn_2Se_2	0	0.577	-0.817	60.5	288.3	13.7	berzelianite $Cu_{2-x}Se$
25	Bn_2Se_2	0.5	0.289	0.817	90.25	266.7	91.3	berzelianite $Cu_{2-x}Se$/umangite Cu_3Se_2
26	Bn_2Se_2	0	-0.577	0.817	60.5	201.7	91.3	berzelianite $Cu_{2-x}Se$/klockmannite CuSe

Table S4. Copper Selenide Syntheses from Additional Screening

Rxn.	Precursor	Time	Temp.	OAm/ODE	Time	Temp.	OAm/ ODE	Phase
		coded	coded	coded	min	˚C	%vol	
1	Ph_2Se_2	-0.513	-0.573	1	30	202	100	weissite $Cu_{2-x}Se$/umangite Cu_3Se_2
2	Ph_2Se_2	1	-0.657	1	120	196.2	100	berzelianite $Cu_{2-x}Se$ /weissite $Cu_{2-x}Se$
3	Ph_2Se_2	-0.513	-0.46	-0.684	30	210.2	20	umangite Cu_3Se_2
4	Ph_2Se_2	-0.932	-0.38	1	5	216.5	100	umangite Cu_3Se_2
5	Ph_2Se_2	-0.933	-0.573	1	5	202	100	umangite Cu_3Se_2
6	Ph_2Se_2	-1	-0.356	1	1	218.3	100	weissite $Cu_{2-x}Se$/umangite Cu_3Se_2
7	Ph_2Se_2	-1	-0.54	1	1	204.5	100	umangite Cu_3Se_2
8	Ph_2Se_2	-1	-0.6	1	1	200	100	weissite $Cu_{2-x}Se$
9	Bn_2Se_2	-0.25	-0.56	-1	45.63	203	5	berzelianite $Cu_{2-x}Se$/klockmannite CuSe
10	Bn_2Se_2	1	-0.89	-0.312	120	178.3	37.7	berzelianite $Cu_{2-x}Se$
11	Bn_2Se_2	-0.03	-0.9	-1	58.73	177.5	5	berzelianite $Cu_{2-x}Se$/klockmannite CuSe
12	Bn_2Se_2	-1	-1	-1	1	170	5	berzelianite $Cu_{2-x}Se$/umangite Cu_3Se_2
13	Bn_2Se_2	-0.77	-1	-1	15	170	5	berzelianite $Cu_{2-x}Se$
14	Bn_2Se_2	-0.51	-1	-1	30	170	5	berzelianite $Cu_{2-x}Se$
15	Bn_2Se_2	0.245	-1	-1	75	170	5	berzelianite $Cu_{2-x}Se$/klockmannite CuSe
16	Bn_2Se_2	0	-1	-1	60.5	170	5	berzelianite $Cu_{2-x}Se$/klockmannite CuSe
17	Bn_2Se_2	-0.26	-1	-1	45	170	5	berzelianite $Cu_{2-x}Se$/klockmannite CuSe
18	Bn_2Se_2	-1	-0.8	-1	1	185	5	berzelianite $Cu_{2-x}Se$
19	Bn_2Se_2	-0.77	-0.8	-1	15	185	5	berzelianite $Cu_{2-x}Se$/klockmannite CuSe
20	Bn_2Se_2	-0.51	-0.8	-1	30	185	5	berzelianite $Cu_{2-x}Se$/klockmannite CuSe

The surrogate model data plotted in **Figure 3** in the main text is visualized differently in **Figure S1**. *ScatteredInterpolant* was used to perform interpolation on the 3-D dataset of scattered surrogate model data. This returns the interpolant (F) for the experimental dataset. Interpolant F can be evaluated at a set of query points, such as (xq, yq, zq) in 3-D, to produce predicted interpolated values $vq = F(xq, yq, vq)$.

Table S5. Coded Phase Combinations and Their Corresponding Colors and Crystal Structures

Code	Color	Phase Combination
A		Berzelianite $Cu_{2-x}Se$
B		Berzelianite $Cu_{2-x}Se$/klockmannite CuSe
C		Umangite Cu_3Se_2/klockmannite CuSe
D		Berzelianite $Cu_{2-x}Se$/umangite Cu_3Se_2
E		Umangite Cu_3Se_2
F		Wurtzite-like $Cu_{2-x}Se$/umangite Cu_3Se_2
G		Wurtzite-like $Cu_{2-x}Se$
H		Weissite-like $Cu_{2-x}Se$/wurtzite-like $Cu_{2-x}Se$
I		Weissite-like $Cu_{2-x}Se$
J		Weissite-like $Cu_{2-x}Se$/umangite Cu_3Se_2
K		Berzelianite $Cu_{2-x}Se$/weissite-like $Cu_{2-x}Se$

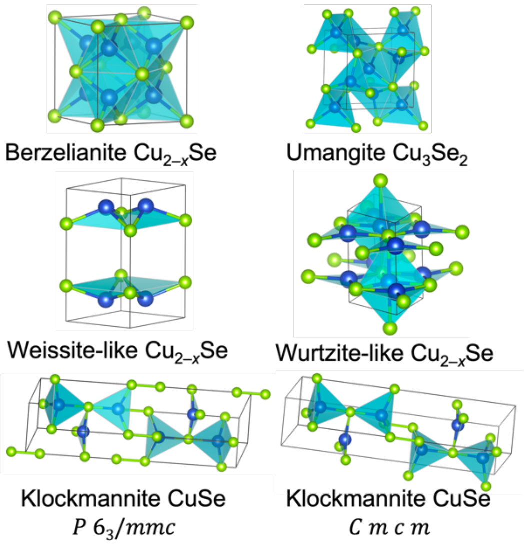

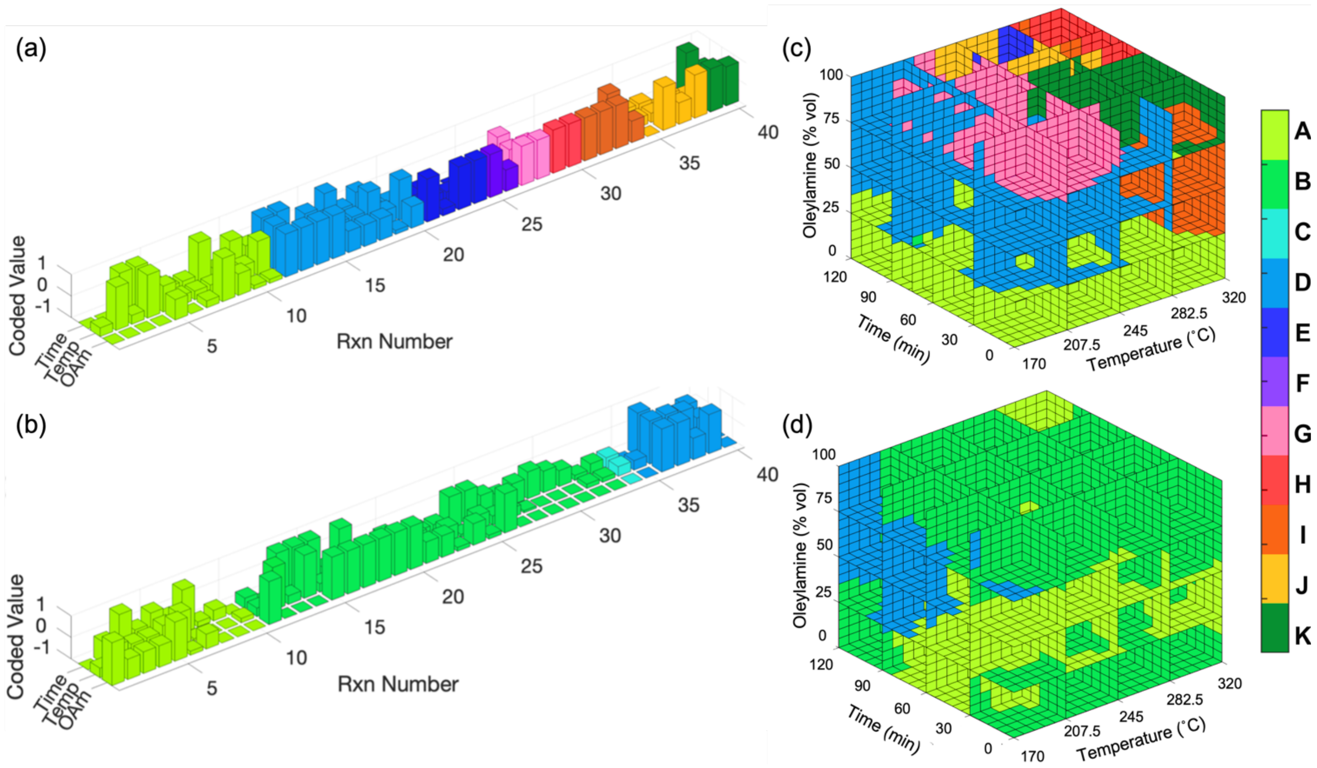

Figure S1. Each reaction in the surrogate model with their respective coded variable values for (a,c) the Ph_2Se_2 precursor and (b,d) the Bn_2Se_2 precursor. (c,d) Phase maps given in **Figure 3** after a 180° rotation. The resulting phase combination for each reaction is indicated by color, with the key given to the right, corresponding to **Table S5**.

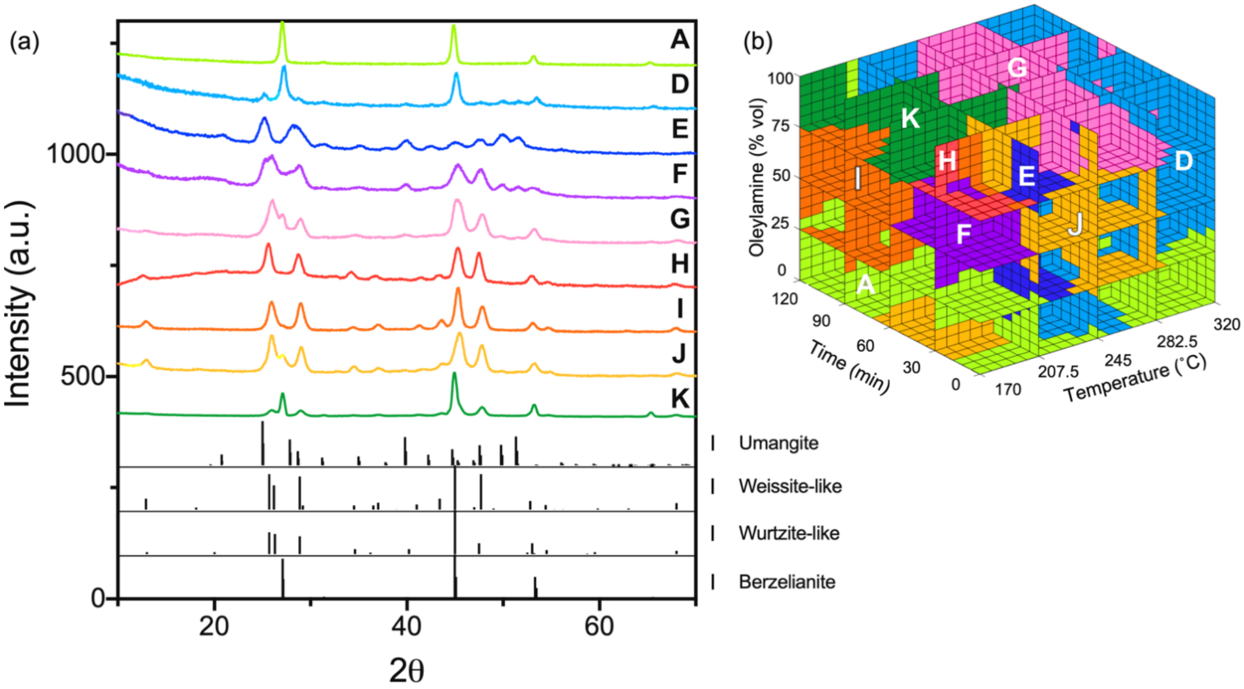

Figure S2. (a) Powder XRD patterns of phase combinations resulting from the Ph_2Se_2 precursor and (b) the corresponding phase map. Coded letters represent the following phase combinations: (A) Berzelianite $Cu_{2-x}Se$ (D) berzelianite $Cu_{2-x}Se$/umangite Cu_3Se_2 (E) umangite Cu_3Se_2, (F) wurtzite-like $Cu_{2-x}Se$/umangite Cu_3Se_2, (G) wurtzite-like $Cu_{2-x}Se$, (H) weissite-like $Cu_{2-x}Se$/wurtzite-like $Cu_{2-x}Se$, (I) weissite-like $Cu_{2-x}Se$, (J) weissite-like $Cu_{2-x}Se$/umangite Cu_3Se_2 and (K) berzelianite $Cu_{2-x}Se$/weissite-like $Cu_{2-x}Se$.

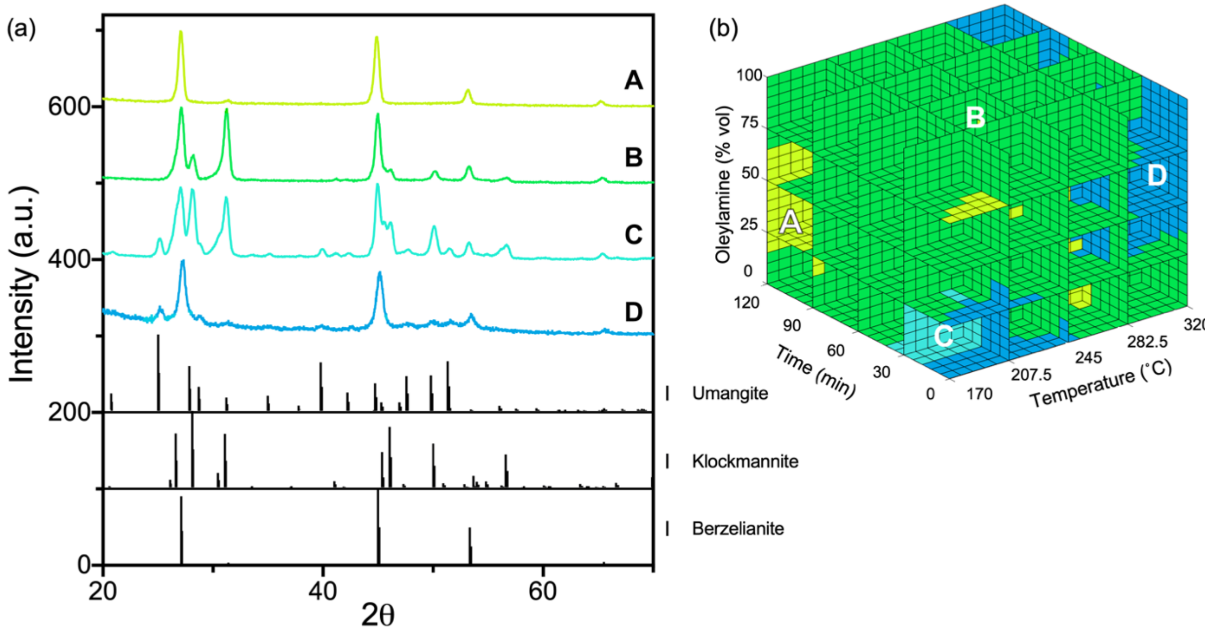

Figure S3. (a) Powder XRD patterns of phase combinations resulting from the Bn_2Se_2 precursor and (b) the corresponding phase map. Coded letters represent the following phase combinations: (A) Berzelianite $Cu_{2-x}Se$ (B) berzelianite $Cu_{2-x}Se$/klockmannite CuSe (C) umangite Cu_3Se_2/klockmannite CuSe and (D) berzelianite $Cu_{2-x}Se$/umangite Cu_3Se_2.

3. Classification

The classification model was chosen by performing a Bayesian optimization of the hyperparameters on the surrogate model data *via* the *fitcauto* function in MATLAB. After 120 iterations the best observed and estimated learner was an ensemble using the bag method over 254 learning cycles, with a minimum leaf size equal to 21 and a maximum number of splits equal to 70. Specifically, bootstrap aggregation, or bagging with random predictor selections at each split (random forest), was used as it was predicted to be best for the multiclass nature of the data. The classification bagged ensemble model was trained with these optimized hyperparameters (as specified by the Bayesian optimization) and leave-one-out cross-validation. This evaluation method was chosen as it is ideal for smaller datasets, providing a much less biased measure of test error compared to using a single test set because we repeatedly fit a model to a dataset that contains n–1 observations.[7] The classification accuracy was 95.7 %, misclassifying only 3 reactions (**Figure S4**). The re-substitution loss was 0.0380, which equates to the misclassification rate. The closer the model predictions are to the observations, the smaller the misclassification error will be, and error ≤ 0.05 is considered acceptable, rendering our model statistically significant.

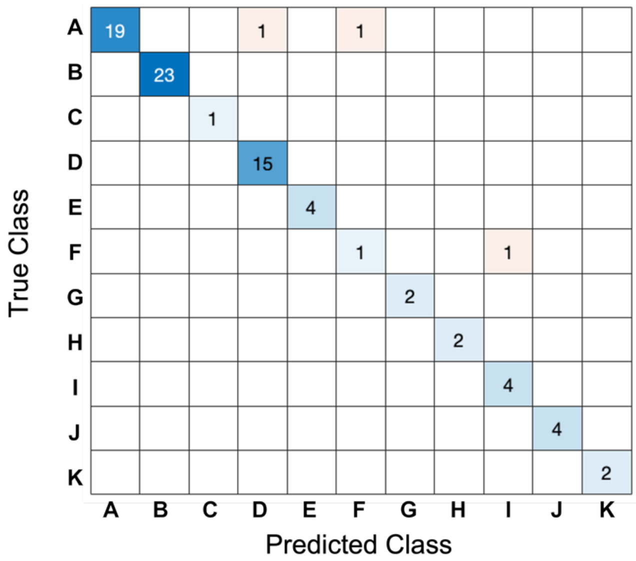

Figure S4. Confusion chart of the classification model predictions, showing correct predictions in blue and incorrect predictions in orange. Letter codes correspond to the legend in **Table S5**.

Other train/test splits were tested, as illustrated in **Figures S5-7**. Considering that some phases were only observed once or twice, and in the case of klockmannite CuSe, not at all, train/test splits that leave out even five reactions can result in a specific phase combination being missed entirely in the training, which makes predictive synthesis of such phases essentially impossible unless further sampling is appended to the initial dataset. For this reason, models trained on the 80 observations in the surrogate model with a holdout > 5% (95/5) almost always lead to errors in

training. A train test split of 97/3 (leaving out 2 observations) resulted in an accuracy of 94.9% with a resubstitution loss of 0.0385 (**Figure S5**). Unsurprisingly, the additional sampling during the optimization throughout the regions where specific phase combinations were scarce in the original dataset enabled the model to maintain a high prediction accuracy over a range of training/testing set ratios. **Figure S6** shows a train test split of 90/10, which resulted in a prediction accuracy of 92.1% and a resubstitution loss of 0.0442. Similarly, a train/test split of 80/20 resulted in a prediction accuracy of 91.9% and a resubstitution loss of 0.050 (**Figure S7**).

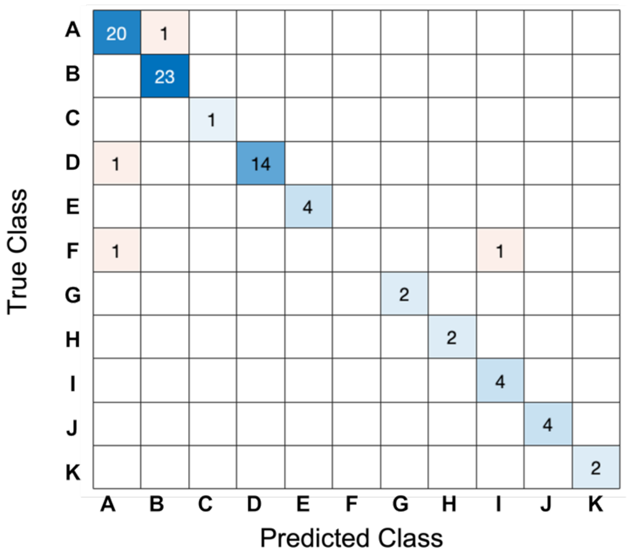

Figure S5. Confusion chart of the classification model predictions using a 97/3 train/test split, showing correct predictions in blue and incorrect predictions in orange. Letter codes correspond to the legend in **Table S5**.

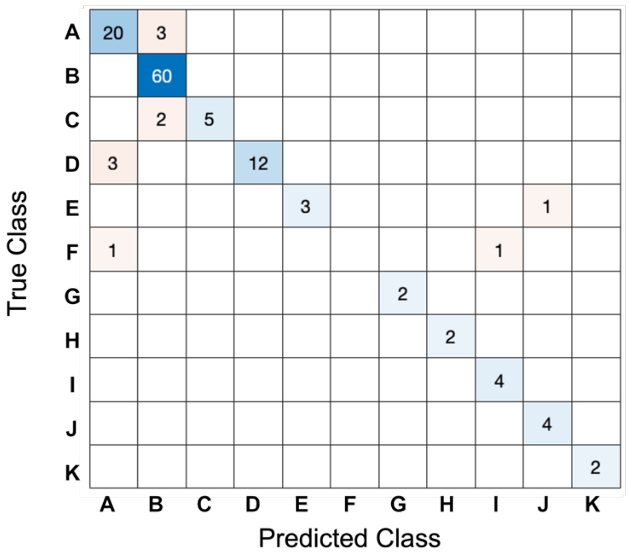

Figure S6. Confusion chart of the classification model predictions using a 90/10 train/test split after the addition of the optimization reactions, showing correct predictions in blue and incorrect predictions in orange. Letter codes correspond to the legend in **Table S5**.

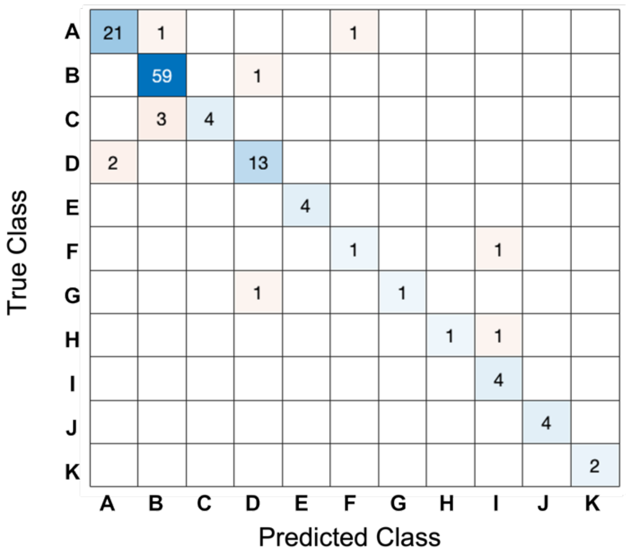

Figure S7. Confusion chart of the classification model predictions using an 80/20 train/test split after the addition of the optimization reactions, showing correct predictions in blue and incorrect predictions in orange. Letter codes correspond to the legend in **Table S5**.

Univariate feature ranking for classification (*fsschi2*) ranks features (predictors) using chi-squared tests. The predictor variables and the response variable (phase) from the training data were provided to a function that returns the indices of predictors ordered by predictor importance. This means the first predictor returned is the most important predictor. The feature rankings are illustrated in **Figure 2** in the main text.

The trained model could then be visualized, which outputted a phase map in the form of a decision tree. Unsurprisingly, the diselenide precursor was the first node on the full decision tree, so the tree was separated by precursor type for simplicity in **Figures S3,4**.

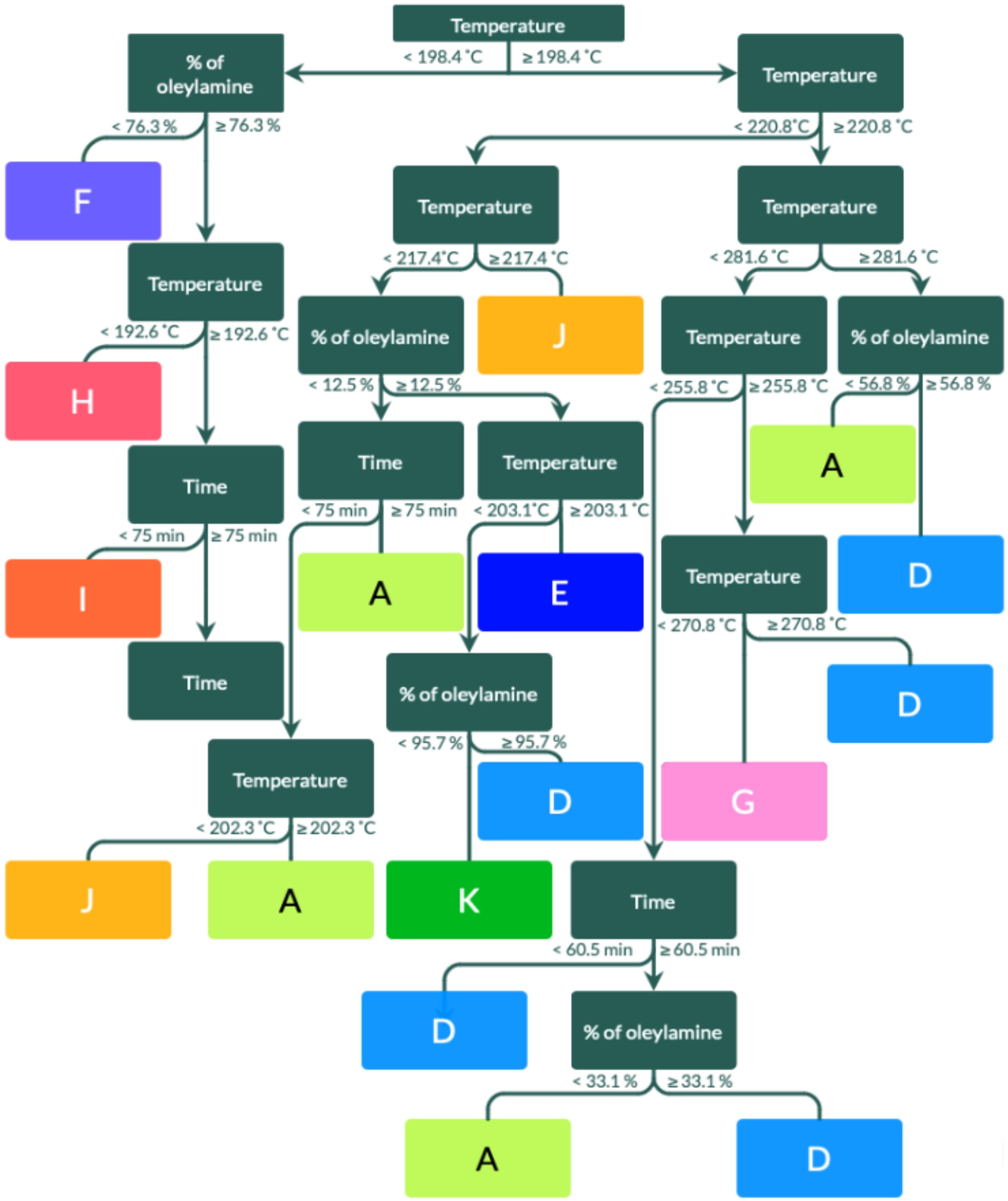

Figure S8. Simplified classification tree for the Ph_2Se_2 precursor, where the letters represent the following phase combinations: (A) berzelianite $Cu_{2-x}Se$, (D) berzelianite $Cu_{2-x}Se$/umangite Cu_3Se_2, (E) umangite Cu_3Se_2, (F) wurtzite-like $Cu_{2-x}Se$/umangite Cu_3Se_2, (G) wurtzite-like $Cu_{2-x}Se$, (H) weissite-like $Cu_{2-x}Se$/wurtzite-like $Cu_{2-x}Se$, (I) weissite-like $Cu_{2-x}Se$, (J) weissite-like $Cu_{2-x}Se$/umangite Cu_3Se_2, and (K) berzelianite $Cu_{2-x}Se$/weissite-like $Cu_{2-x}Se$.

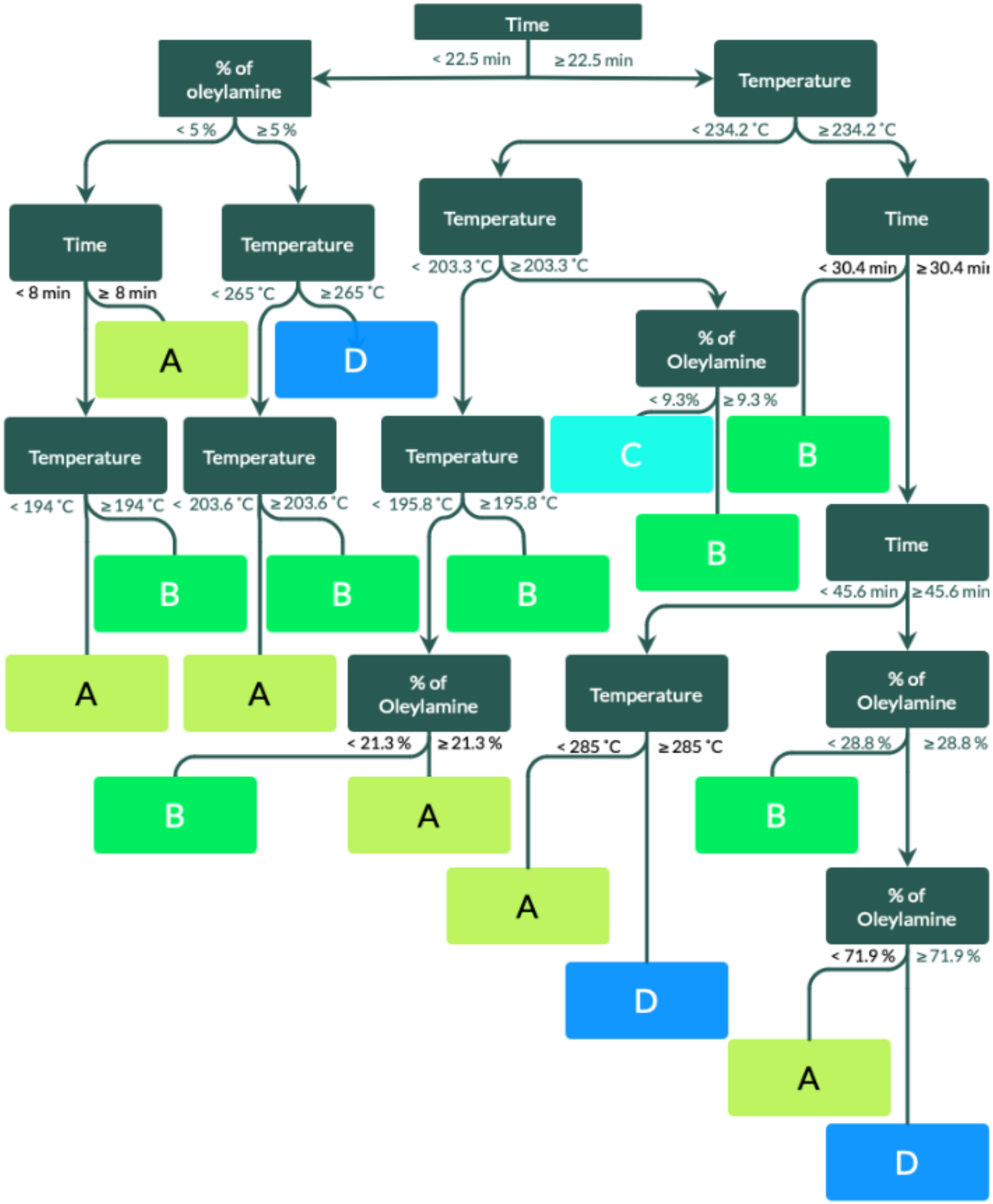

Figure S9. Simplified classification tree for the Bn_2Se_2 precursor, where the letters represent the following phase combinations: (A) berzelianite $Cu_{2-x}Se$, (B) berzelianite $Cu_{2-x}Se$/klockmannite CuSe, (C) umangite Cu_3Se_2/klockmannite CuSe, (D) berzelianite $Cu_{2-x}Se$/umangite Cu_3Se_2.

4. Isolating the Target Klockmannite Phase

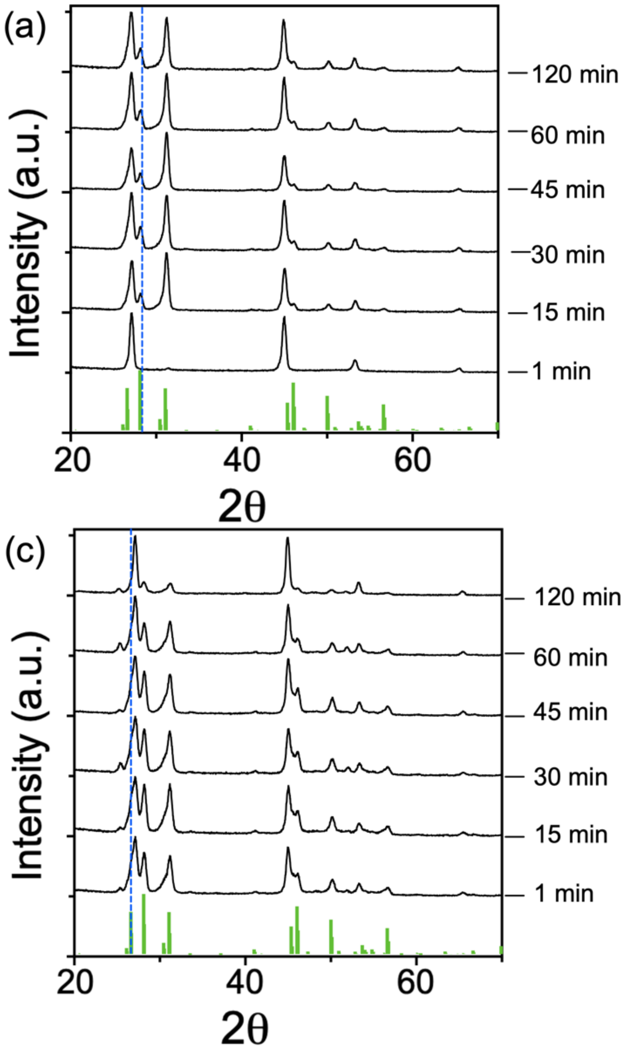

Figure S10. Powder XRD patterns for initial temperature screening of klockmannite CuSe phase synthesized with Bn_2Se_2 as a function of time at (a) 205, (b) 215, and (c) 225 ˚C. Reference klockmannite CuSe stick pattern is indicated in green.

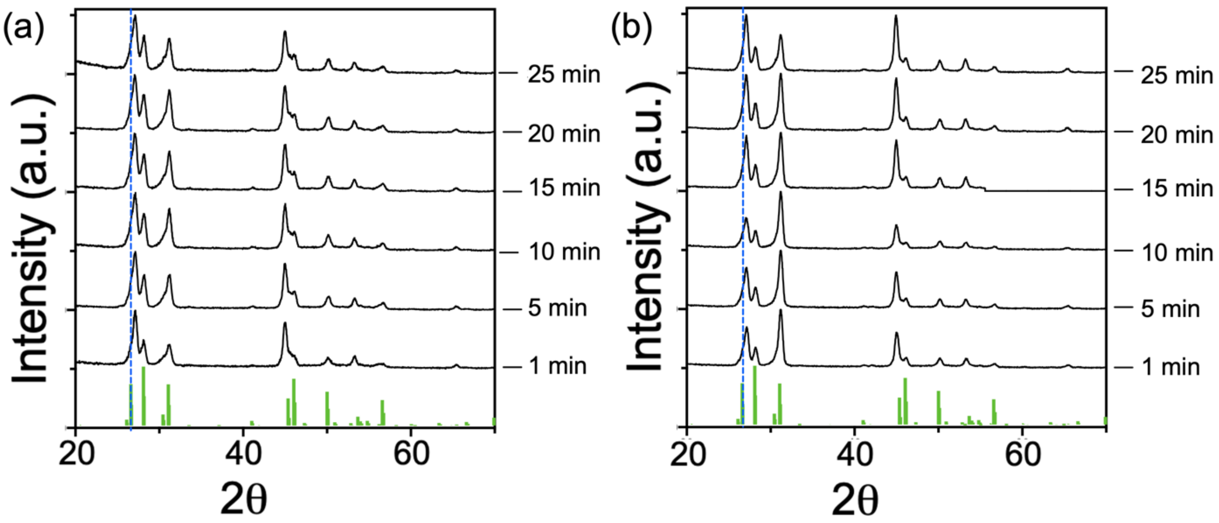

Figure S11. Powder XRD patterns for the second iteration of temperature screening of klockmannite CuSe phase synthesized with Bn_2Se_2 as a function of time at (a) 220 and (b) 230 ˚C. Reference klockmannite CuSe stick pattern is indicated in green.

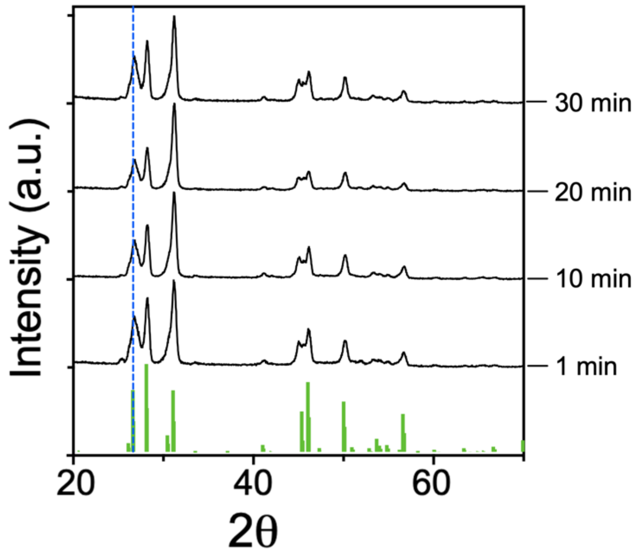

Figure S12. Powder XRD patterns from aliquot study of klockmannite CuSe phase synthesized with Bn_2Se_2 over time at 223.5 ˚C. Reference klockmannite CuSe stick pattern is indicated in green.

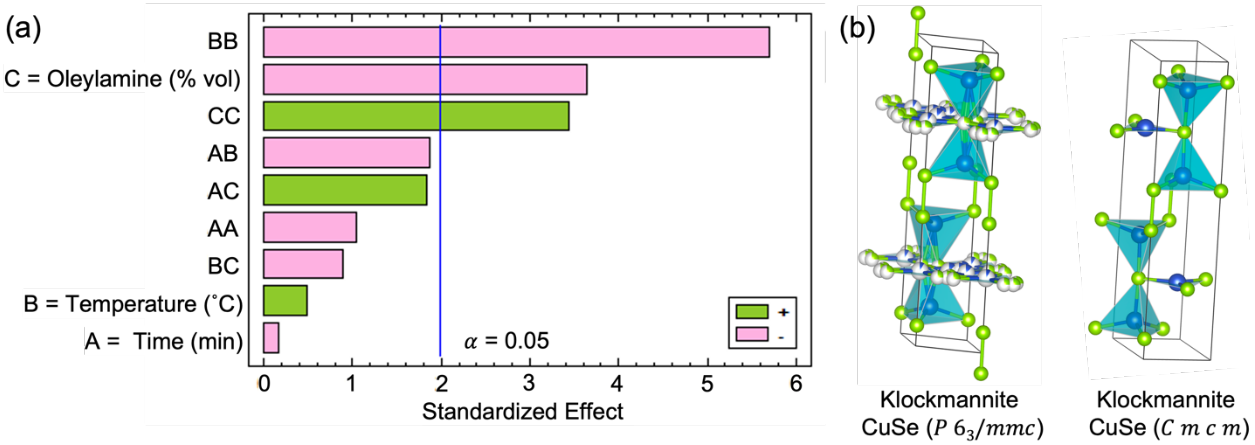

Figure S13. (a) Pareto chart of the quantified percentage of klockmannite CuSe, showing the significance of the variables and their interaction effects. The blue line indicates 95% confidence interval. (b) Crystal structures of the two klockmannite polymorphs.

5. References

(1) Chakrabarti, D. J.; Laughlin, D. E. The Cu−Se (Copper-Selenium) System. *Bull. Alloy Phase Diagr.* **1981**, *2*, 305–315.

(2) Glazov, V. M.; Pashinkin, A. S.; Fedorov, V. A. Phase Equilibria in the Cu-Se System. *Inorg. Mater.* **2000**, *36*, 641–652.

(3) Okamoto, H. Supplemental Literature Review of Binary Phase Diagrams: Ag-Ca, Al-Yb, As-Fe, B-Zr, Co-U, Cu-Se, Cu-Th, La-Mo, Mg-Sn, Mo-Th, Sn-Ta, and Te-Ti. *J. Phase Equilibria Diffus.* **2017**, *38*, 929–941.

(4) Liu, S.; Zhang, Z.; Bao, J.; Lan, Y.; Tu, W.; Han, M.; Dai, Z. Controllable Synthesis of Tetragonal and Cubic Phase Cu_2Se Nanowires Assembled by Small Nanocubes and Their Electrocatalytic Performance for Oxygen Reduction Reaction. *J. Phys. Chem. C* **2013**, *117*, 15164–15173.

(5) Gariano, G.; Lesnyak, V.; Brescia, R.; Bertoni, G.; Dang, Z.; Gaspari, R.; De Trizio, L.; Manna, L. Role of the Crystal Structure in Cation Exchange Reactions Involving Colloidal Cu_2Se Nanocrystals. *J. Am. Chem. Soc.* **2017**, *139*, 9583–9590.

(6) Hernández-Pagán, E. A.; Robinson, E. H.; La Croix, A. D.; Macdonald, J. E. Direct Synthesis of Novel $Cu_{2-x}Se$ Wurtzite Phase. *Chem. Mater.* **2019**, *31*, 4619–4624.

(7) Wong, T.-T. Performance Evaluation of Classification Algorithms by K-Fold and Leave-One-out Cross Validation. *Pattern Recognit.* **2015**, *48*, 2839–2846.
